# Cave *Thiovulum* (*Candidatus* Thiovulum stygium) differs metabolically and genomically from marine species

**DOI:** 10.1038/s41396-022-01350-4

**Published:** 2022-12-17

**Authors:** Mina Bizic, Traian Brad, Danny Ionescu, Lucian Barbu-Tudoran, Luca Zoccarato, Joost W. Aerts, Paul-Emile Contarini, Olivier Gros, Jean-Marie Volland, Radu Popa, Jessica Ody, Daniel Vellone, Jean-François Flot, Scott Tighe, Serban M. Sarbu

**Affiliations:** 1 Leibniz Institute for Freshwater Ecology and Inland Fisheries, IGB, Dep 3, Plankton and Microbial Ecology, Zur Alte Fischerhütte 2, OT Neuglobsow, 16775 Stechlin, Germany; 2 Berlin-Brandenburg Institute of Advanced Biodiversity Research (BBIB), Berlin, Germany; 3 “Emil Racoviţă” Institute of Speleology, Clinicilor 5-7, 400006 Cluj-Napoca Romania, Romania; 4 Center for Electron Microscopy, “Babeș-Bolyai” University, Clinicilor 5, 400006 Cluj-Napoca, Romania; 5 Institute of Computational Biology, University of Natural Resources and Life Sciences, Gregor-Mendel-Straße 3, 31180 Vienna, Austria; 6 Department of Molecular Cell Physiology, Faculty of Earth and Life sciences, De Boelelaan 1085, 1081 HV Amsterdam, The Netherlands; 7 Institut de Systématique, Evolution, Biodiversité (ISYEB), Muséum National d’Histoire Naturelle, CNRS, Sorbonne Université, EPHE, Université des Antilles, 97110 Pointe-à-Pitre, France; 8 Laboratory for Research in Complex Systems, Menlo Park, CA USA; 9 Department of Energy Joint Genome Institute, Lawrence Berkeley National Laboratory, 94720 Berkeley, CA USA; 10 River Road Research, 62 Leslie St, Buffalo, NY 1421 USA; 11 Evolutionary Biology and Ecology, Université libre de Bruxelles (ULB), C.P. 160/12, Avenue F.D. Roosevelt 50, 1050 Brussels, Belgium; 12 Vermont Integrative Genomics Lab, University of Vermont Cancer Center, Health Science Research Facility, Burlington, Vermont, VT 05405 USA; 13 Interuniversity Institute of Bioinformatics in Brussels—(IB)², Brussels, Belgium; 14 “Emil Racoviţă” Institute of Speleology, Frumoasă 31-B, 010986 Bucureşti, Romania; 15 Department of Biological Sciences, California State University, Chico, CA 95929 USA

**Keywords:** Water microbiology, Biogeochemistry, Phylogenetics, Bacterial genomics, Microbial ecology

## Abstract

*Thiovulum* spp. (Campylobacterota) are large sulfur bacteria that form veil-like structures in aquatic environments. The sulfidic Movile Cave (Romania), sealed from the atmosphere for ~5 million years, has several aqueous chambers, some with low atmospheric O_2_ (~7%). The cave’s surface-water microbial community is dominated by bacteria we identified as *Thiovulum*. We show that this strain, and others from subsurface environments, are phylogenetically distinct from marine *Thiovulum*. We assembled a closed genome of the Movile strain and confirmed its metabolism using RNAseq. We compared the genome of this strain and one we assembled from public data from the sulfidic Frasassi caves to four marine genomes, including *Candidatus* Thiovulum karukerense and *Ca*. T. imperiosus, whose genomes we sequenced. Despite great spatial and temporal separation, the genomes of the Movile and Frasassi Thiovulum were highly similar, differing greatly from the very diverse marine strains. We concluded that cave *Thiovulum* represent a new species, named here *Candidatus* Thiovulum stygium. Based on their genomes, cave *Thiovulum* can switch between aerobic and anaerobic sulfide oxidation using O_2_ and NO_3_^-^ as electron acceptors, the latter likely via dissimilatory nitrate reduction to ammonia. Thus, *Thiovulum* is likely important to both S and N cycles in sulfidic caves. Electron microscopy analysis suggests that at least some of the short peritrichous structures typical of *Thiovulum* are type IV pili, for which genes were found in all strains. These pili may play a role in veil formation, by connecting adjacent cells, and in the motility of these exceptionally fast swimmers.

## Introduction

*Thiovulum* spp. are spherical bacteria typically <25 µm in diameter [1], but some can reach up to 50 µm [2]. *Thiovulum* spp. are mostly known from marine environments [3], with few old descriptions from freshwater [4, 5]. Described species are sulfur-oxidizing chemolithoautotrophs [6], some of which display an extremely fast motility [7, 8]. *Thiovulum* cells typically form veils close to surfaces [1, 9] and can attach to these using a secreted stalk [10]. They are normally located close to the oxic-anoxic interface near sediments or microbial mats [1, 11, 12], where the planar organization of the veil and the rapid movements of the cells’ flagella produce a convective transport of O_2_ [13].

**Fig. 1:**
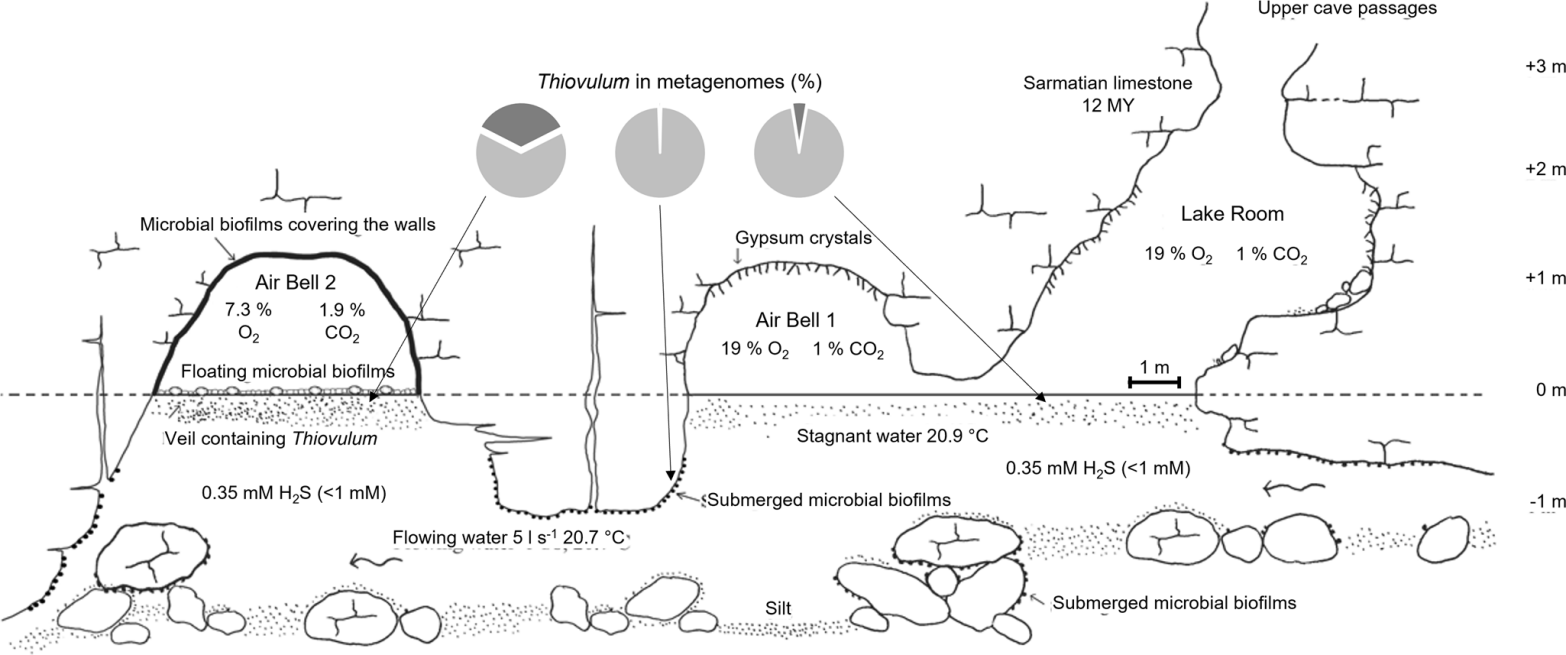
Longitudinal profile of the sampling area in Movile Cave ((modified after [101, 102]. The microbial community containing *Thiovulum* cells (depicted here as dots present at, and beneath the water surface) was sampled in the Lake Room, and in Air Bell 2 alongside submerged microbial mats from Air Bell 1. *Thiovulum* 16 S rRNA gene made up to a maximum of 5%, 0.9%, and 35% of the 16 S rRNA genes retrieved from metagenomic samples (dark gray in pies) from these cave sections, respectively. More details on community composition are presented in Fig. S1. The scale bar refers to the length of the rooms, which are all *ca*. 1 m in width.

Movile Cave is located near the town of Mangalia, SE Romania (43°49’32“N, 28°33'38“E), 2.2 km inland from the Black Sea shore. It consists of a 200 m long upper dry passage that ends in a small lake allowing access to a 40 m long, partially submerged, lower cave level (Fig. 1). Thick and impermeable layers of clays and loess cover the limestone in which the cave is developed, preventing input of water and nutrients from the surface [14]. Sulfidic groundwater flows constantly at the bottom of Movile Cave’s lower passages. Because of the morphology of the lower cave passages (Fig. 1) and a slight difference in water temperatures, the water near the surface is practically stagnant. Riess et al. [15] determined that oxygen penetrates only the upper 0.8 mm of the water column, below which the water is anoxic.

Cave ecosystems are normally characterized by stable conditions and provide a window into subsurface microbiology [16] In the absence of natural light, these ecosystems are typically fueled by chemolithoautotrophy via the oxidation of reduced compounds such as H_2_S, Fe^2+^, Mn^2+^, NH_4_^+^, CH_4_, and H_2_. Most of the microbiological studies performed in Movile Cave, as summarized in Kumaresan et al. [17], are based on samples of microbial biofilms floating on the water surface or covering rock surfaces in the Lake Room, Air Bell 1, and in Air Bell 2 where the atmosphere is low in O_2_ (*ca*. 7%) and enriched in CO_2_ (*ca*. 2%) and CH_4_ (1–2%) [18]. The microbial communities at these sites were found to include sulfur oxidizing bacteria such as *Beggiatoa*, *Sulfurospirillum*, *Thiobacillus*, *Thiomonas*, *Thioploca*, *Thiothrix*, and *Thiovirga* [19–21] methylotrophs such as *Methylomonas*, *Methylococcus* and *Methylocystis* [22], *Methylotenera*, *Methylophilus*, and *Methylovorus* [19, 20]; ammonia and nitrite oxidizers such as *Nitrosomonas, Nitrospira* and *Nitrotoga* [20] and the methanogenic *Archaea Methanobacterium* [23] and *Methanosarcina* [24]. Of these, activity was confirmed only for a few taxa [20, 22–25] while for others it was inferred based on phylogenetic association.

**Fig. 2:**
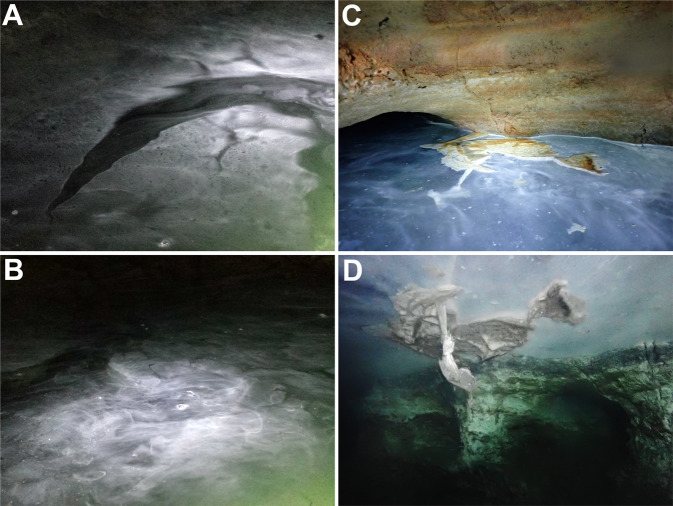
Images of surface veils from the Lake Room (**A** and **B**) and Air Bell 2 (**C** and **D**) in Movile Cave. See also Supplementary Video 1. **C**, **D** are images of the same location taken above (**C**) and below (**D**) the water surface, showing the thin *Thiovulum* veil. The images are *ca*. 1 m wide.

In the lower level of Movile Cave, at and directly below the water surface (<3 mm), we observed a loose floating veil (Fig. 2 and Supplementary video 1). Using genetic and microscopic analyses, we found that this agglomeration of bacteria is dominated by a species of the genus *Thiovulum* (Fig. 1, S1 and results). Using a comparative genomic approach, we compare the metabolic potential of this newly identified freshwater strain and another cave *Thiovulum* sp. we assembled from public data to their marine counterparts, including the genomes of *Candidatus* Thiovulum karukerense [26] and *Ca*. T. imperiosus [2], whose genomes are also presented here.

## Materials and methods

The general structure of the Movile Cave and sampling locations are depicted to scale in Fig. 1. Briefly, the lake in the Lake Room is *ca*. 1 m wide and 3 m long, Air Bell 1 is of similar dimensions, and Air Bell 2 is *ca*. 1 m wide by 5 m long. The water depth in the cave is *ca*. 1.5 m. The ceiling in Air Bell 2 is at about 1 m above the water surface.

Movile Cave was sampled, in most cases, from within the water with as minimal disturbance possible. Specifically, Air Bell 2 can only be reached by SCUBA diving. There, the diver stayed only so long as necessary to collect the samples and document the environment. Air bubbles ascending from the dive likely disturbed the surface resulting in the patchy appearance of the floating microbial mats (Fig. 2). Prior to disturbance by the sampling team, the microbial veils covered the entire water surface, as could be evaluated from underwater while entering Air Bell 2 and by observing side sections of the chamber that are unaffected by the divers.

### Molecular analyses

Unless stated otherwise, samples of water were collected into sterile tubes from the surface (upper few mm) of the Lake Room and from the Air Bells (Fig. 1), targeting specifically the white veil.

### gDNA

For total DNA extraction a total of 12 (four tubes each from the Lake Room, Air Bell 2, and submerged mats in Air Bell 1), 50-mL water samples were collected in July 2019, and preserved, on site (Lake Room) with ethanol to a final concentration of 50%. The samples were then transferred to −20 °C within several hours of fixation where they were held till further treatment.

Microorganisms from each water sample (25 mL water +25 mL ethanol) were concentrated separately using a vacuum pump and Nalgene single-use analytical filter funnels (Thermo Fisher Scientific, MA, USA), with the included filter replaced with a 0.2 µm isopore membrane filter (Millipore Sigma, MA, USA). Prior to filtration, the glass assembly components were autoclaved at 121.5 °C for 30 min wet and 20 min dry at 1.4 bar (20 psi). Filters were placed into 50 µL conical tubes with 300 µL 1× PBS, 1.5 µl 2% azide, and a sterile scalpel blade. Samples were minced using an OMNI bead ruptor elite (OMNI International, GA, USA) on a 4 ms^−1^ 30 s program. The resulting sample was centrifuged to collect the microorganisms separated from the filters and extracted for gDNA using a modified version of the Omega Bio-Tek Universal Metagenomics kit protocol (OMEGA Bio-Tek, GA, USA). Fifteen µL of MetaPolyzyme (Millipore Sigma, MA, USA) was added to each sample and incubated at 35 °C for 13 h followed by three cycles of freeze and thaw alternating between 80 °C for 2 min and a −80 °C freezer for 10 min. Further digestion was subsequently performed by adding 35 µL Proteinase K (Omega Bio-Tek, GA, USA) and incubated at 55 °C for 1 h. Following complete enzymatic digestion, the sample was extracted using the manufacturer’s protocol (Omega Bio-Tek Universal metagenomics kit). Briefly, 500 µL ML1 buffer (CTAB) was added the digested sample and incubated at 55 °C for 15 min One volume of Tris-stabilized (pH > 7.5) phenol-chloroform-isoamyl alcohol mix (25:24:1) was used for purification and the resulting upper aqueous phase was removed and combined with RBB (guanidinium*)* buffer and 100% ethanol and applied to a DNA silica column supplied with the kit. Final DNA was eluted in 35 µL of elution buffer. DNA was quantified using a Qubit spectrofluorometer and Nanodrop ND-1000 (ThermoFisher Waltham MA USA).

### Thiovulum aggregates

In September 2019, samples were also collected for targeted “single-cell” sequencing of *Thiovulum*. For this, water samples were fixed on-site (1:1 v:v) in self-made RNAlater (4 M (NH_4_)_2_SO_4_; 15 mM EDTA (from 0.5 M, pH 8.0 stock); 18.75 mM Na-citrate (from 1 M stock)). The samples were then transferred to 4 °C (to prevent freezing) within several hours of fixation where they were held till further treatment.

Ten individual *Thiovulum* aggregates were picked under a binocular at 50X magnification, after which cells lysis and DNA amplification were carried out by three initial cycles of freeze-thawing with liquid N_2_ and a 65 °C thermal block and subsequently the lysis and amplification protocol of the REPLI-g Single-Cell DNA amplification kit, following the manufacturer instructions (Qiagen, Hilden, Germany). Of the ten amplified aggregates, four were selected for nanopore library preparation (see below).

### Total RNA

In August 2021, 8 additional tubes were collected for RNA extraction and preserved on-site with ethanol to a final concentration of 50%. The samples were then transferred to −20 °C within several hours of fixation where they were held till further treatment.

Total nucleic acids were extracted from polycarbonate filters (Millipore, 0.2 µm pore size) upon which microorganisms from 50 mL of ethanol-fixed water samples were collected. A total of six samples were extracted, three from the Lake Room and three from Air Bell 2. Extraction was done following Nercessian et al. [27] with minor modifications. In brief, a CTAB extraction buffer containing SDS and N-laurylsarcosine was added to the samples together with an equal volume of phenol/chloroform/isoamylalcohol (25:24:1) solution. The filter samples were subject to bead-beating (FastPrep-24 5 G Instrument, MP Biomedical, Eschwege, Germany), followed by centrifugation (14,000 g), a cleaning step with chloroform, and nucleic acid precipitation with PEG-6000 (Sigma-Aldrich, Taufkirchen, Germany). The precipitated nucleic acids were rinsed with 1 mL of 70% ethanol, dried and dissolved in water. DNA was digested by two sequential treatments with the TurboDNAfree Kit (Invitrogen Thermo Fisher Scientific, Dreieich, Germany) following the manufacturer’s instructions. DNA removal was evaluated using a PCR for 16 S rRNA gene. First strand cDNA was then generated using the High-Capacity cDNA Reverse Transcription Kit (Applied Biosciences, Thermo Fisher scientific), and was sent for sequencing at the Core Genomic Facility at RUSH University, Chicago, IL, USA.

### Other *Thiovulum* spp.

Samples of *Ca*. T. karukerense [26] and *Ca*. T. imperiosus [2] were respectively obtained from veils developing above the sediment and on bones deployed in marine mangrove in Guadeloupe (Lesser Antilles). Samples were collected manually between March and May 2021 in ~1 m depth (16°16'32.7“N 61°33'28.5“W). While dense veils of *Ca*. T. karukerense naturally form above the mangrove sediment throughout the year, we had to deploy cleaned pig bones as described before [2] to collect live *Ca*. T. imperiosus individuals. The veil formation of this species depends on the anaerobic degradation of lipids from bone-marrow by marine sulfate reducing bacteria which produce high concentrations of hydrogen sulfide. *Candidatus* T. imperiosus veils usually appear after 8 to 10 days of deployment. Both marine veils were collected manually using distinct 60 mL syringes, brought to the laboratory. The *Ca*. T. karukerense sample was processed for DNA extraction within 1 h as described in the next section. The sample of *Ca*. T. imperiosus contained several hundreds of cells easily distinguishable under a microscope by their large size (~50 µm in diameter). Single *Ca*. T. imperiosus cells were isolated under a stereomicroscope and processed for single-cell genomics as described in the next section.

### Shotgun metagenomic sequencing (Illumina and Oxford Nanopore)

Shotgun metagenomic sequencing of DNA from Movile Cave was accomplished using both Illumina and Oxford Nanopore sequencing technologies. For Illumina sequencing, 1 ng of genomic DNA from each sample was converted to whole-genome sequencing libraries using the Nextera XT sequencing reagents according to the manufacturer’s instructions (Illumina, San Diego CA). Final libraries were checked for library insert size using the Agilent Bioanalyser 2100 (Agilent Technologies Santa Clara, CA) and quantified using Qubit spectrofluorometry. The final sample was sequenced using paired end 2 x 150 sequencing on an MiniSeq (Illumina) system.

A first pass of Oxford Nanopore sequences was obtained using the SQK LSK109 ligation library synthesis reagents on a Rev 9.4 nanopore flow cell with the GridION X5 MK1 sequencing platform, resulting in a total of 131.8 Mbp of reads with a N50 of 1.3 kbp.

Oxford Nanopore sequencing was additionally performed on DNA amplified from individual *Thiovulum* aggregates. Libraries for Nanopore sequencing (four in total) were prepared using the LSK-108 kit following the manufacturer’s protocol using 1 µg of DNA but skipping the size selection step. The prepared libraries were loaded on two MIN106 R9 flow cells, generating a total of 5.7 Gbp of reads with a length N50 of *ca*. 3.7 kbp. Basecalling of all Oxford Nanopore reads was performed using Guppy 4.0.11.

A DNA library for *Ca*. Thiovulum imperiosus was prepared from a single cell, visually identified based on its large diameter. It was isolated under the stereomicroscope and washed twice in 0.1 µm filtered seawater. Seawater was removed and the cell was stored at −80 °C until further processing. The cell was thawed, and we amplified the genomic DNA by multiple displacement amplification using the REPLI-g kit (Qiagen). A DNA library was created from 200 pg of DNA from the amplified product using Nextera XT DNA library creation kit (Illumina).

A DNA library for *Ca*. Thiovulum karukerense was prepared from a DNA extraction performed on thousands of cells pipetted under a stereomicroscope and transferred into 0.1 um filtered seawater. Cells were then transferred to a second vial containing new 0.1 µm filtered seawater. To limit contaminations to a minimum, this step was repeated three more times. Cells were pelleted and genomic DNA was extracted with the DNeasy kit (Qiagen) following the manufacturer’s protocol. DNA libraries were created from 200 pg of DNA using Nextera XT DNA library creation kit (Illumina). Both libraries were sequenced on an Nextseq High Output (Illumina) platform.

### cDNA sequencing

The provided single stranded cDNA was adjusted to 45 µL with water and sheared with the Rapid Shear gDNA shearing kit (Triangle Biotechnology, Durham, NC, USA). Briefly, 5 µL of Rapid Shear Reagent was added to the bottom of a 24-well PCR plate. cDNA (45 µL) was then added to the well and the plate was sealed and held on ice till shearing. Shearing took place for 5 min in a sonication bath. The resulting DNA of about 350 bp in length, was used directly in the Swift 1 S protocol (Accel-NGS 1 S Plus kit, Swift Biosciences, Ann Arbor, Mi, USA). The resulting sheared DNA was adjusted to 5 ng/µL, and a total of 75 ng was used for library prep, except for sample LR1 (Lake Room) that had low cDNA concentration. For this sample, the maximum volume of 15 µL was used as input. Library prep was as per the Swift Protocol with 6 cycles of PCR during indexing. Following library prep, all libraries were pooled in equal volume by combining 2 µL of each library for a final bead clean up with 0.85X AmpPure beads (Beckman Coulter Life Sciences, Indianapolis, IN, USA). This QC pool was then sequenced on an MiniSeq MO flow cell (Illumina). The resulting index distribution was used to re-pool the libraries for an SP flow cell (Illumina) sequencing run with sample LR1 pooled at maximum volume available.

Sequencing data generated in this study were deposited in NCBI Sequence Read Archive. Data from Movile Cave is available under bioproject accession number PRJNA673084. The genome of *Thiovulum* sp. from Frasassi is available under bioproject accession number PRJNA846597. The genomes of *Ca*. T. imperiosus, and *Ca*. T. karukerense are available under bioproject accession number PRJNA830902.

### Metagenomic data analysis

To obtain information on relative *Thiovulum* abundance in Movile Cave, the raw short-read libraries (metagenomic) were analyzed with phyloFlash (V 3.3; [28]) that provides the relative abundance and taxonomic annotation of 16 S rRNA genes from metagenomic libraries.

To assemble the genome of the Movile Cave *Thiovulum*, nanopore reads were assembled using Flye 2.8.1-b1676 [29] with default parameters. The resulting assembly graph (GFA) was examined using Bandage [30], allowing to delineate a set of high-coverage (>300X) contigs against a background of low-coverage (<100X) contigs. To verify that these high-coverage contigs corresponded to *Thiovulum*, the published proteome of *Thiovulum* ES [11] was aligned on the GFA using tblastn [31] within Bandage (parameters: minimum identity 70%, minimum coverage 70%), revealing that nearly all tblastn hits were concentrated on the high-coverage contigs and vice-versa. The GFA was therefore pruned to retain only the high-coverage contigs, which were all interconnected. The remaining 31 contigs were exported as FASTA then scaffolded using SLR [32]; the nine resulting scaffolds were mapped back to the GFA to resolve most repeats, and the remaining repeats were resolved manually until obtaining a circular genome. A final polishing step was performed with unicycler-polish from Unicycler v0.4.9b [33] using the complete set of Illumina reads (for a total depth of coverage of 12X of the genome) and the subset of Nanopore reads longer than 5 kb (coverage *ca*. 50X). Polishing consisted of two cycles of pilon 1.23 [34], one cycle of racon 0.5.0 [35] followed by FreeBase [36], then 30 additional cycles of short-read polishing using pilon 1.23, after which the assembly reached its best ALE score [37].

### *Thiovulum* sp. genome assembly from public databases

To obtain genomic information from additional cave-dwelling *Thiovulaceae* we downloaded all available metagenomic libraries from the Frasassi caves in Italy (SRR10997432, SRR1559028, SRR1559230, SRR1559353, SRR1560064, SRR1560266, SRR1560848, SRR1560849, SRR1560850, SRR8191123, SRR8194889, SRR8197024, SRR8200784, SRR8202337, SRR8203764). The short read libraries were quality-trimmed using Trimmomatic [38] and scanned for the presence of *Thiovulum* 16 S rRNA gene using PhyloFlash [28], revealing that library SRR1560850 contained >170,000 reads of *Thiovulum* sp. 16 S rRNA gene. A metagenomic assembly of library SRR1560850 was therefore conducted using Megahit [39], after which the assembly was binned using Metabat2 [40]. The obtained bins were taxonomically annotated using the GTDB-TK tool [41] resulting in one *Thiovulum* bin. The phylogenetic tree generated by the GTDB-TK tool from a single-copy marker gene multilocus alignment suggested that the Movile and Frasassi caves *Thiovulum* genomes were closely related, hence, both genomes were used to recruit all *Thiovulum* related reads from all Frasassi libraries. The obtained reads were re-assembled, binned and taxonomically annotated as above to obtain a more complete assembly.

### Genome quality assessment, annotation, and comparison

The completeness of the *Thiovulum* genomes obtained was assessed using CheckM [42]. The continuity of the Movile *Thiovulum* genome was evaluated using the unicycler-check module in Unicycler v0.4.9b. Annotation of all genomes was performed using the command-line Prokka [43] and DRAM [44] tools as well as the KEGG [45], EggNOG 5.0 [46], PATRIC [47, 48], and RAST [49, 50] annotation servers. A COG [51] analysis was done using the ANVIO tool [52]. OperonMapper [53] was used to inspect the organization of genes into operons in the circular Movile Cave genome. CRISPRs where identified using CRISPR finder tool [54]. Metabolic models of the annotated genomes were calculated using PathwayTools (V25.5) [55] using the RAST annotation of each genome as the model basis. Components of type IV pili systems were identified using MacSyFinder [56] using precomputed models [57]. Average Nucleotide Identity (ANI) and Average Amino-acid Identity (AAI) were calculated using FastANI [58] and EzAAI [59], respectively.

### 16 S rRNA gene phylogenetic trees

A maximum-likelihood phylogenetic tree was calculated, including only 16 S rRNA gene sequences longer than 1200 nt, using FastTree 2 [60] using all *Thiovulum* sequences in the SILVA REF (V138.1) database (*n* = 71), three 16 S rRNA gene sequences obtained from the assembled genome of the Movile Cave *Thiovulum* (see below) and sequences of several different *Sulfurimonas* species as an outgroup. The tree was calculated using the generalized time-reversible model and the discrete gamma model with 20 rate categories. Local support values were calculated with the Shimodaira-Hasegawa test.

### Transcriptomic analysis

The six libraries containing cDNA sequences (three from Air Bell 2 and three from Lake Room) were quality-trimmed using trimommatic [38] and mapped against the complete genome of the Movile Cave *Thiovulum* sp. using Salmon (version 1.6) [61]. Ribosomal RNA data were removed from the mapping results and TPM (Transcripts Per Kilobase Million) values were recalculated to reflect mRNA expression. Further removal of tRNA sequences did not alter the results. The RNA data was analyzed using the iDEP (v. 0.95) online tool [62] that provides an online graphical user interface for the DeSEQ2 [63] and Limma [64] packages for RNAseq analysis. Differential expression was considered significant with a 2-fold difference and a false discovery rate smaller than 0.1. Taxonomic composition of the active community was obtained by analyzing the 16 S rRNA gene from the transcriptomic read libraries using PhyloFlash as above (V 3.3; [28]). Viral transcripts were identified using VirSorter2 (v.1.1) [65], annotated against the NCBI viral refseq database [66] release 209 using BLAST and quantified using Salmon version 1.6 [61].

### Water chemistry

Temperature, pH, and TDS (total dissolved solids) were measured using an Extech 341350A-P Oyster Series pH/Conductivity/TDS/ORP/Salinity Meter (FLIR System, Nashua, NH, USA) in 2019 and with a Hanna Multiparameter HI9829 (Hanna Instruments Inc., Woonsocket, RI, USA) in 2020.

Measurements of NH_4_^+^, SO_4_^2-,^ NO_2_^-^, NO_3_^-^ were obtained as part of the cave monitoring conducted by some of the co-authors and were analyzed using ion chromatography as previously described [67, 68]

### Cell enumeration and Electron microscopy and elemental analysis

For cell enumeration, two 15-mL water samples from the Lake Room were collected from outside of the water to avoid disturbance by divers and preserved on-site with formaldehyde to a final concentration of 4%. *Thiovulum* cell enumeration was done on a 96-grid fields 5-mL chamber microscopy slide using a light microscope (Olympus BX51, Olympus, Tokyo, Japan). Cell counts were averaged from 15-grid squares.

For transmission electron microscopy, two unpreserved 15-mL samples collected from the water surface in Movile Cave were centrifuged, and the microorganisms were resuspended and fixed for 2 h with 2.7% glutaraldehyde in phosphate buffered saline (1× PBS). The cells were then rinsed three times in 1× PBS, and finally fixed for 1 h with 2% osmic acid in 1× PBS. The cells were harvested again by centrifugation, dehydrated in graded acetone-distilled water dilutions, and embedded in epoxy resin. Sections of about 100 nm thickness were produced with a diamond knife (Diatome, Hatfield) using a Leica UC6 ultramicrotome (Leica Microsystems, Wetzlar, Germany) and were stained with lead citrate and uranyl acetate (Hayat, 2001). The grids were examined with a Jeol JEM transmission electron microscope.

Five samples were collected for scanning electron microscopy (SEM) and bright-field scanning transmission electron microscopy (BFSTEM). These were fixed on site with 2.7% glutaraldehyde in 1× PBS from concentrated stocks. The samples were transported to the microscopy center within 24 h where they were air dried in the lab on 0.22 µm mesh-sized Millipore filters, without further handling or pre-concentration step. Samples were sputter coated with 10 nm gold and examined for SEM on a JEOL JSM 5510 LV microscope (Jeol, Japan), and for BFSTEM on a Hitachi SU8230 (Tokyo, Japan).

Energy-dispersive X-ray spectroscopy (EDX) analysis was performed using an EDX analyzer (Oxford Instruments, Abingdon, UK) and with the INCA 300 software.

## Results

### Field observations

A pale-white veil, with a vertical thickness of 2 to 3 mm, was observed at and below the water surface in Movile Cave (Fig. 2), resembling microbial veils described for sulfur-oxidizing bacteria [7, 68]. Nevertheless, in Movile Cave, this agglomeration of cells did not form slime or a strongly cohesive aggregation. By contrast, the samples used to assemble the Frasassi caves *Thiovulum* genome were obtained from stream biofilms [69].

### Water chemistry

Physico-chemical parameters of the Movile Cave water, as measured in the Lake Room (Table 1 and Supplementary Table 1) resembled those from previous studies of the cave [18] and revealed minimal differences between water sampled at 1 m depth (water inlet) and near-surface water.

**Table 1:** Physico-chemical parameters of the water in the Lake Room of Movile Cave at 1 and 0.1 m depths.

	TDS mg L^−1^	pH	T °C	NH_4_^+^ µM	NO_2_^−^ µM	NO_3_^−^ µM	SO_4_^2−^ µM	H_2_S µM
1 m (~inflow)	584–985	7.5–8.0	24	18	ND	19	24	285
0.1 m		7.1–7.4	20.5–21.4	62	0.43	22	32	363

### Microscopy

**Fig. 3:**
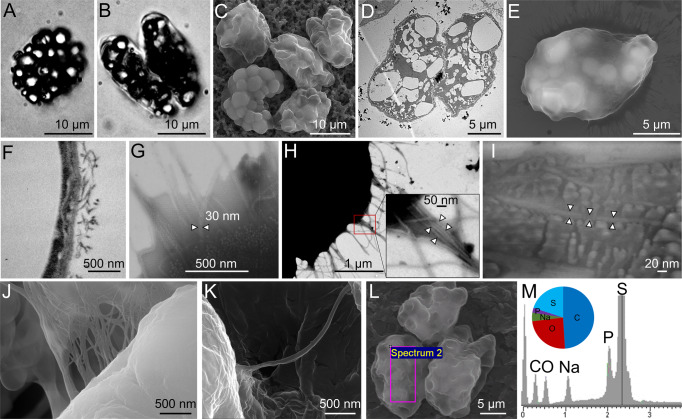
Light and electron microscopy of *Ca*. Thiovulum stygium strain Movile. Optical images of giant globular cells colonizing the subsurface veil from Movile Cave (**A**, **B**) including a cell undergoing division (**B**). Each cell carries 20 to 30 sulfur inclusions (**C**) and large bright spots in **A**, **B**. TEM images of *Thiovulum* show the cellular localization of sulfur inclusions of various shapes and sizes (**D** white spots). Ovoid cells divide along their long axis (**B**, **D**). The region where the cell membrane is not fully closed between dividing cells is marked with three black arrows (**D**). The cell is densely covered in short filamentous structures (**E**) protruding from the outer membrane (**F**). These structure with an average diameter of ca. 30 nm (**G**), are bundles of thinner filaments (**H**) with a diameter of 9–12 nm (**H**, **I**). The filaments often appear to connect *Thiovulum* cells one to another (**J**, **K**). EDX analysis on *Thiovulum* cells (**L**) inspected under SEM show the typical elemental composition of the cells (**M**) and confirm the high sulfur content of the internal globules. Note that the height of the peaks in the EDX spectra do not correlate with the element*’*s ratio but with the X-ray signal intensity. **A**, **B** transmitted light micrographs. **C**, **J**–**L** Scanning Electron Micrographs. **D**, **F** transmission electron micrographs. **E**, **G**–**I** scanning transmission electron micrographs.

A veil similar to that seen in the Lake Room (Fig. 2A, B) occurred in Air Bell 1 and was even more pronounced in Air Bell 2 (Fig. 2C, D). In Air Bell 2 the veil consisted of large, spherical to ovoid, bacterial cells (Fig. 3A, B) fitting the description of the genus *Thiovulum*. These cells had a diameter of 12–16 µm, contained 20–30 sulfur globules each (Fig. 3A–C), and occurred in the Lake Room at densities of approximately 5.5 × 10^3^ cells/mL. Transmission Electron Microscopy (TEM) observations showed that these large cells were Gram-negative (Fig. 3D–F) and confirmed the existence of 20–30 irregularly shaped sulfur inclusions within each of the cells (Fig. 3D). Light and TEM imaging revealed *Thiovulum*-like cell divisions along the long cell axis (Fig. 3B, D). Short peritrichous filamentous appendages (Fig. 3E, F) observed on the surface of the cells resemble those noticed earlier on other *Thiovulum* species [6]. These filaments measured <3 µm in length (Fig. 3E) and between 20–30 nm in diameter (Fig. 3G). Closer analysis revealed that at least some of the larger structures consisted of bundles of thinner filaments (Fig. 3H) with diameters of 9–12 nm each (Fig. 3H, I). Scanning electron microscopy (SEM) revealed that some of these filaments connected the cells via multiple or single threads (Fig. 3J, K). Energy-dispersive X-ray (EDX) analysis (Fig. 3L, M) confirmed that the intracellular globules contained sulfur (20.9–26.1%), along with elements common in organic matter such as carbon (49–49.2%) and oxygen (21.1–24.6%), and a few other elements in low abundance such as sodium (2.4–3.4%) and phosphorus (1.2–2.2%).

### Phylogenetic identification and relative abundance of *Thiovulum* sequences

*Thiovulum* was found in highest abundance (sequence frequency) in Air Bell 2 (<35%), followed by Lake Room (<5%) and submerged microbial mats (<0.9%) (see pie charts in Fig. 1). A detailed community composition based on 16 S rRNA genes obtained from the metagenomic libraries is presented in Supplementary Fig. 1. *Thiovulum* rRNA sequences made up >94% both in the Lake Room and in Air Bell 2 (Supplementary Fig. 1), revealing its dominance in the active microbial community in both cave compartments at the time of sampling.

**Fig. 4:**
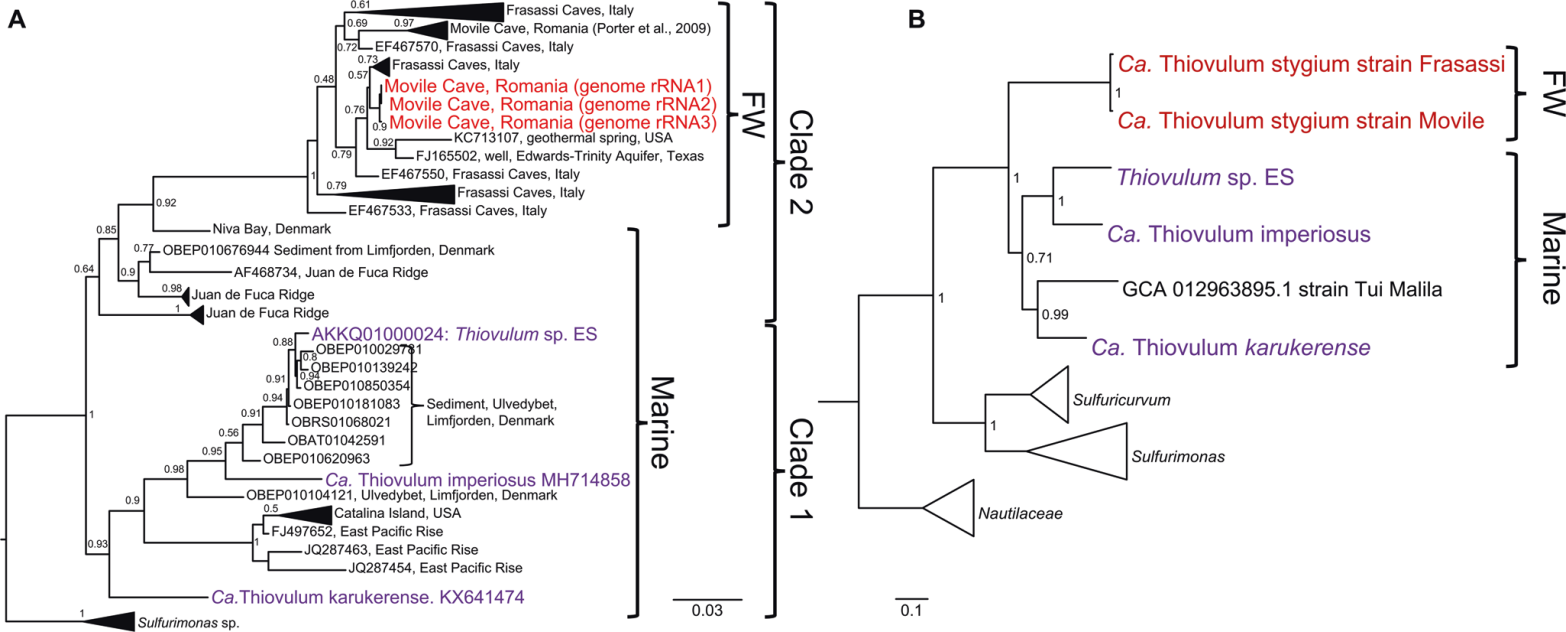
Phylogenetic analysis of *Thiovulum* spp. Maximum-likelihood placement of the 16 S rRNA gene of *Thiovulum* spp. available in the SILVA SSU database (V138.1 [103]) alongside those from the cave (*Ca*. Thiovulum stygium) and marine *Thiovulum* genomes introduced in this study (**A**) whose phylogenomic placement based on a concatenated protein alignment is presented in **B**. The protein alignment was generated using GTDB-TK [41]. The Shimodaira-Hasegawa local support values (ranging from 0 to 1) are shown next to each node. Abbreviation “st.” in figure refers to strain.

The 16 S rRNA gene sequences obtained from the closed genome of the Movile Cave *Thiovulum*, alongside other *Thiovulum* sequences obtained from Movile Cave in an earlier study [70], formed a separate clade together with other cave and subsurface, freshwater *Thiovulum* spp., specifically from the sulfidic Frasassi caves in Italy (Fig. 4A). This clade belonged to one of two clades, termed here 1 and 2, of otherwise marine *Thiovulum* spp., the other one of which included *Thiovulum* ES, for which a draft genome is available [11] as well as the 16 S rRNA gene sequences of *Ca*. T. imperiosus [2] and *Ca*. T. karukerense [26].

**Fig. 5:**
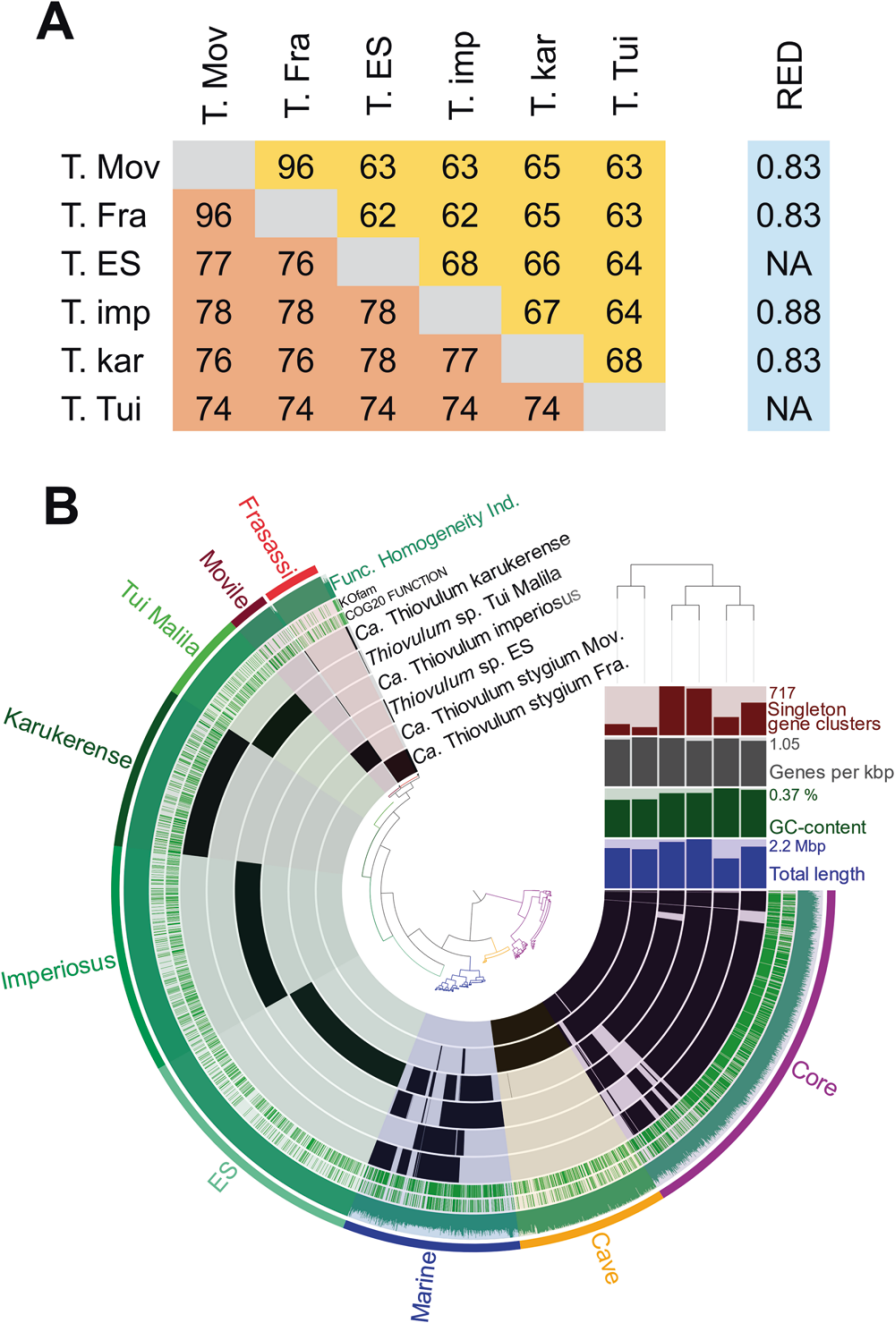
Genome comparison of *Thiovulum*spp. Average Nucleotide Identity (**A** lower triangle), Average Amino-acid Identity (**A** Upper triangle) among the 6 *Thiovulum* strains, the relative evolutionary divergence (RED) value (**A** right column), and an overall comparison of the same genomes (**B**). The black bars represent the presence absence of a COG function in each genome. RED values were obtained from the GTDB-TK [41] tool for the newly inserted genomes. The term *Candidatus* was omitted from the names of *Thiovulum* spp. due to space considerations.

A phylogenetic tree was constructed from a multilocus alignment of single-copy marker genes from the marine *Thiovulum* spp.: *Thiovulum* ES [11], *Ca*. T. imperiosus, *Ca*. T. karukerense, and a public assembly (GCA_012963895.1) from the Tui Malila hydrothermal plume classified as *Thiovulum* [71], along the freshwater genome of *Thiovulum* from Movile Cave, a metagenome assembled genome from the Frasassi caves, and multiple groups of *Campylobacterales*. The tree confirms the existence of two *Thiovulum* clades, and the separation of the freshwater Frasassi and Movile *Thiovulum* genomes from the marine strains. The latter also show a higher diversity among the four marine *Thiovulum* spp. (Fig. 4B). This is further seen in the high Average Nucleotide Identity (ANI) and average Amino Acid Identity (AAI) between the two cave strains as compared to their dissimilarity from the marine *Thiovulum* spp. and the dissimilarity of those among each other. (Fig. 5A). Both AAI and ANI analyses revealed that while the Movile and Frasassi strains are highly similar (96% AAI and ANI), they are equally distant from the marine strains as those exhibit ANI values ranging between 74–78% and AAI between 62–68%.

### Genome analysis

Of the four genomes assembled in this study, only the assembly of metagenomic data from Movile Cave resulted in a closed circular genomic sequence (Fig. S2). The size of the assembled genomes ranged between 1.72–2.20 MBP and GC content varied between 28–37% (Table 2). Genome completeness was estimated using CheckM [42] ranging between 90–96% and evalG (as incorporated into the PATRIC server (now BV-BRC); [72]) ranging between 95.8% and 100% with the exception of *Thiovulum* sp. Tui Malila which was evaluated at 66% completeness. Using a *Campylobacteraceae* specific set of marker genes did not improve the completeness prediction. The marker genes that could not be identified are listed in Supplementary Table 2. Of these 9 genes/domains (PF00308.13, PF02464.12, PF04085.9, PF08299.6, PF08459.6, PF12344.3, TIGR00615, TIGR02124, TIGR03423) could not be found in any of the 6 genomes and additional 4 genes/domains were missing from both freshwater genomes (TIGR00196, TIGR00539, TIGR00121, PF03853.10). All genomes were analyzed using different tools with the results summarized in Supplementary Datasets 1–5.

**Table 2:** Genome quality assessment.

							CheckM			PATRIC			
	Strain	Scaff.	Size MB	CDs	N50 kb	L50	Compl. (%)	Con. (%)	Het. (%)	Compl. (%)	Con. (%)	CC	FC
Freshwater	*Ca*. Thiovulum stygium strain Movile GCA_021582295.1	1	1.72	1887	–	–	93.2	0	0	95.8	0	94.3	93.3
	*Ca*. Thiovulum stygium strain Frasassi JAODIV000000000	127	1.80	1848	32.9	18	94.4	0.4	25	100	0	93.5	93.5
Marine	*Thiovulum* sp. strain ES GCA_00026965.1	206	2.08	2190	28.1	23	96.1	2.6	0	100	4	95.1	93.4
	*Ca*. Thiovulum imperiosus JAMJTW000000000	36	2.20	2225	10.8	7	95.9	1.0	66	100	0	95.6	94.8
	*Ca*. Thiovulum karukerense JAMJTX000000000	191	1.86	1876	12.4	45	93.3	3.2	16	100	7.7	94.7	91.6
	*Thiovulum* sp. strain Tui Malila GCA_012963895.1	178	1.34	1436	10.2	39	90.4	1.0	66	66	11.1	93.5	92.3

*Compl* Genome completeness, *Con* Contamination, *Het* Strain heterogeneity - analyzed using single copy marker genes, *Scaff* Number of Scaffolds in the final assembly, *CDs* Number of coding genes, *CC and FC* Coarse and Fine consistency as evaluated by EvalG [72]

#### Carbon metabolism

**Fig. 6:**
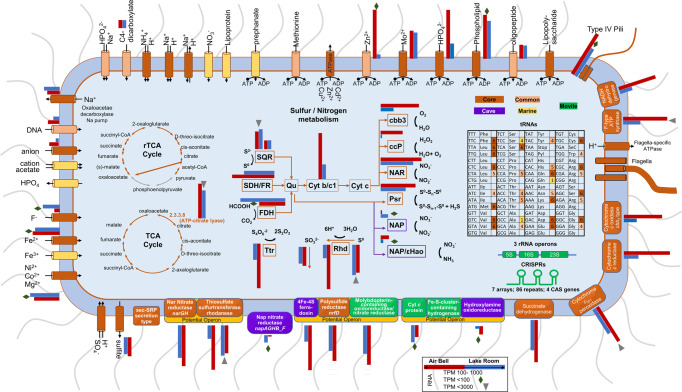
Graphical summary of main components of the freshwater and marine *Thiovulum* strains. Elements or reactions are colored differently whether they occur only in cave strains (purple; both genomes), marine strains (yellow; at least two of four strains), and common to both environments (peach; at least one cave and one marine, genome). Core elements or reactions are colored in brown. Green elements are unique to the Movile Cave genome. Gray arrows in the reductive TCA (rTCA) cycle show missing reactions. The sulfur/nitrogen metabolism model was drawn based on Grote et al. [104], Hamilton et al. [69], and Poser et al. [84]. SQR sulfide-quinone oxidoreductase, SDH/FR succinate dehydrogenase/fumarate reductase, FDH formate dehydrogenase, Qu quinone, Cyt *b/c*1 quinone cytochrome oxidoreductase, *cbb*_3_ cytochrome *c* oxidase, ccp cytochrome *c* peroxidase, NAP periplasmic nitrate reductase, NAR membrane bound nitrate reductase, Psr polysulfide reductase, εHao Epsilonproteobacterial hydroxylamine oxidoreductase, Ttr tetrathionate reductase, Rhd rhodanese-related sulfurtransferase. CRISPRs were identified in all genomes with the Movile strain data depicted here. Comparative gene expression between *Thiovulum* in Air Bell 2 (red) and the Lake Room (blue) are shown for each gene depicted where the TPM value was above 10. Genes were the TPM value was below 100 or above 1000 are marked with a diamond and inverse triangle, respectively. For proteins consisting of multiple subunits, the expression is the genes encoding for one of the subunits. For SQR and RhD expression is shown for both copies of the genes.

Similar to published *Thiovulum* ES, all genes required for C fixation via the reductive TCA cycle were identified. in the new freshwater and marine *Thiovulum* genomes. The oxidative TCA cycle was found to be complete as well in all the *Thiovulum* species with the citrate synthase gene (EC 2.3.3.1) replaced by ATP-citrate lyase. (EC 2.3.3.8) (Fig. 6, Supplementary Figs. 3–8, Supplementary Dataset 1–6).

#### Sulfur metabolism

All annotation approaches (Supplementary Datasets 1–6) revealed only few genes involved in dissimilatory sulfur cycling, including two versions of the sulfide:quinone oxidoredutase (Type VI and IV) that oxidizes sulfide to polysulfide, and the polysulfide reductase gene *nrfD* that carries out the reverse process. In the Movile strain, *nrfD* was found in a 3-gene potential operon together with the large subunit of the assimilatory nitrate reductase (*narB*) and the *ttrB*; tetrathionate reductase subunit B. Rhodanese sulfur transferase proteins were identified in all genomes. In the Movile strain these were located in a 6-gene operon containing two other subunits of a nitrate reductase (*narH* and *narG*). As for the two other genes in this operon, one is related to cytochrome *c* and the other could not be annotated (G2Y-810). The *tauE* sulfite exporter was identified in all genomes as well, in the Movile strain genome as part of a 5-gene operon involved in the transport of molybdate (*modCABD*). Sulfite dehydrogenase, dissimilatory sulfite reductase (*dsrAB*), the sox genes or adenylyl sulfate reductase (*aprAB*) that carry out the sulfide oxidation to SO_4_^2-^ could not be found by any of the annotation tools nor by manual BLAST against all sequences available for each of those proteins in the UniProt database.

#### Nitrogen metabolism

The membrane-bound nitrate reductase (*nar*) found also in *Thiovulum* ES, was identified in all *Thiovulum* spp. genomes. The Movile and Frasassi strains possess in addition also periplasmatic nitrate reductases encoded by the *nap* genes encoded in one operon (*napAGHB_F*). A hypothetical protein encoded in this operon in the Movile genome is likely part of the *napF* gene (G2Y-1099) as seen by BLAST analysis in other *Campylobacteraceae*. The gene for hydroxylamine dehydrogenase, which is often encountered in genomes from *Campylobacterota* [73] (formerly referred to as *Epsilonproteobacteria* [74]), was also identified in the Movile strain genome as part of a 3-gene operon including two unannotated hypothetical genes.

#### Chemotaxis and motility

*Thiovulum* spp. are highly motile bacteria, therefore, we inspected motility and chemotaxis genes. All genes necessary for flagellar assembly were found in all *Thiovulum* genomes. The chemotaxis genes, *cheA*, *cheW*, *cheD*, and *cheY* were identified as well as additional *che*Y-like domains. The c*heB* gene was found only in the Movile strain (G2Y-1843), and *cheX* was missing from *Thiovulum* spp. strains karukerense and ES. The *cetA* and *cetB* (G2Y-174:176 *cetABB*’) energy taxis genes were identified in the Movile strain while all other genomes contained only *cetB* in 1 or 2 copies. The homolog of the *Escherichia coli* aerotaxis (*aer*) gene was found in the genomes of *Ca*. T. imperiosus and of the Movile strain. Additionally, multiple methyl-accepting chemotaxis proteins were identified in each genome.

Genes for type IV pili assembly and secretion were identified in all genomes. While some of these genes were identified by the classical annotation tools, other were recognized among the “hypothetical proteins” using HMM search against various families of type IV pili [57]. The various genes make up all the necessary genes for type IV pili, yet their closest relatives in the database come from different families of type IV pili, e.g., T4Pa and T4Pb.

### Transcript analysis

**Fig. 7:**
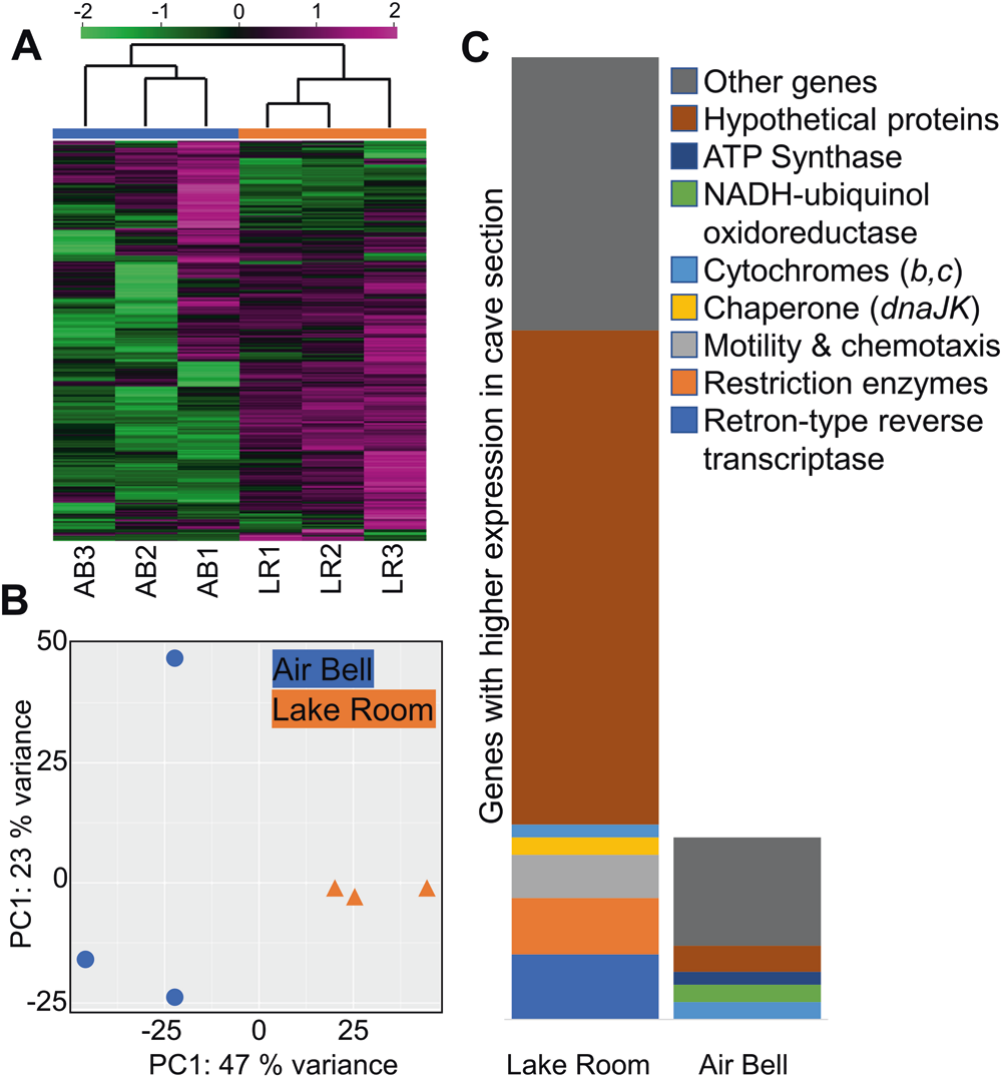
Comparison of mRNA transcriptomic profiles of the *Ca*. Thiovulum stygium strain Movile obtained from triplicates samples collected in Air Bell 2 and in the Lake Room. A heatmap shows that the two environments are separated from one another with clusters of genes expressed more, or less in one of the two environments (**A**). The values in the heatmap are log-transformed TPM values and normalized using each gene’s standard deviation. Principle components analysis (**B**) demonstrates the separation between samples mainly across PC1 likely representing sample location. Differential expression analysis (**C**) revealed that 222 genes are significantly more expressed in the Lake Room as compared to Air Bell 2, whereas 48 genes. are significantly less expressed.

To confirm that *Thiovulum* is transcriptionally active in Movile Cave, as well as to assess which genes of the predicted metabolic pathways are transcribed, samples from Air Bell 2 and from the Lake Room of Movile Cave were collected for RNA analysis. 16 S rRNA transcripts of *Thiovulum* dominated all samples, making up more than 94% of the active community (Fig. S1) even though *Thiovulum* DNA was rare in the Lake Room in our DNA samples. Despite the similarity in relative rRNA transcript abundance, the gene expression profiles differ significantly between the two sites (Fig. 7). In both the heatmap (Fig. 7A) and the principal component analysis (Fig. 7B), the samples from the different environments clustered separately, with clear clusters of genes differently expressed in the two cave compartments. Differential expression analysis (Fig. 7C; Supplementary Dataset 1) revealed that 222 genes were more expressed in the Lake Room compared to Air Bell 2, while the opposite comparison resulted in 42 genes. In contrast, the TPM normalized expression of the housekeeping genes *rpoB*, *gyrB*, and *bipA* was similar in both compartments, therefore, confirming that the differential expression of genes is not a methodological bias. Over half of the genes more expressed in the Lake Room encoded for hypothetical proteins to which no function could be assigned. Retron-type reverse transcriptases were the most dominant group of genes (*n* = 15) also exhibiting some of the highest transcription level with TPM values up to 19,000. Genes over expressed in samples from Air Bell 2 were related to energy generation including cytochromes c and b as well as F-type ATP synthase. The entire gene expression data is available in Supplementary Dataset 1 and is additionally depicted in Fig. 6 next to the displayed genes or functions.

## Discussion

We investigated the morphological, phylogenetic, and genomic aspects of a planktonic *Thiovulum* species that belongs to a newly recognized clade of freshwater *Thiovulum*. We were able to identify, and assemble, another genome of a freshwater *Thiovulum* from public metagenomic data from the Frasassi caves. We further compared these two genomes from freshwater clade to the genomes of four marine *Thiovulum* spp.: *T*. sp. ES [11] and *T*. sp. GCA_012963895.1 (Tui Malila) [71], retrieved from public databases, and the genomes of *Ca*. T. imperiosus [2] and *Ca*. T. karukerense [26] whose genomes are presented here for the first time. The main aspects of this comparison are depicted in Fig. 6 and in more details in Supplementary Dataset 7. Supplementary Figs. 3–8 offer a detailed, Pathway-Tools automatic- reconstruction of the pathways detected in each of the six genomes.

### Phylogeny

Cave *Thiovulum* (Movile and Frasassi caves) are phylogenetically distinct from marine species. This is evident in both genomic and 16 S rRNA gene phylogenetic analyses for which more sequence data were available. The rRNA gene analysis also highlighted that, all sequences obtained from sulfidic caves, or subsurface environments (e.g., a drinking water well) formed a freshwater subclade in the otherwise marine Clade 2, suggesting a transition of these species from a marine to a freshwater environment. The degree of genetic diversity of the marine genomes was higher in comparison to the freshwater ones, as supported by the ANI and AAI analyses. This phenomenon could be a result of the freshwater cave *Thiovulum* spp. being isolated in stable subsurface environments and, therefore, diverging less from their ancestors [75]. Nevertheless, this should be further evaluated as more freshwater and marine *Thiovulum*, species become available for comparison. In another case of marine-freshwater transitions, it was also evidenced that freshwater *Pelagibacteraceae* evolved slower than their marine counterparts, revealing highly similar strains across large geographical distances [76] similar to the Movile and Frasassi *Thiovulum* strains.

Core marine genes (KEGG orthologs or COG) could be identified; however, these do not functionally differentiate marine from freshwater *Thiovulum*. Similarly, we could not detect differences in copy numbers of genes related to osmoregulation. As such, given the phylogenetic indication that the freshwater clade emerged from the marine *Thiovulum*, it may be that the osmoregulation genes occurring in the marine genomes, are either necessary in the cave water, or were not yet lost by the freshwater *Thiovulum* spp.

We propose that the two cave strains belong to the same *Thiovulum* species while the other 4 genomes represent 4 different *Thiovulum* species. In absence of cultures, to properly evaluate and define new bacterial species, we rely on molecular analyses such as ANI, AAI, and RED. Despite the great difference in genomic sequence (ANI analysis), the similarity in amino acids sequences (AAI > 62%) leads us to conservatively suggest that the investigated genomes belong all to the genus *Thiovulum* rather than different genera within the *Thiovulaceae* family [77]. The obtained RED values for the 4 new species whose genomes are presented here are between 0.83–0.88. Given the clustering of all *Thiovulum* genomes together, these values are in good agreement with a genus level separation as seen in Parks et al., [78] and the GTDB (v207) RED statistics report (https://gtdb.ecogenomic.org/stats/r207). This is also supported by a K/θ analysis [79–81] where the species separation limit was defined as K/θ > 6 (corresponding to ca. 99% species separation probability: Fig. S10). Accordingly, we propose naming the cave *Thiovulum* sp. as *Candidatus* Thiovulum stygium (sp. nov.). Stygium, referring to the mythical underworld river Styx, appropriately describes the aquatic underground biome of cave *Thiovulum*.

### Sulfur and nitrogen metabolism

*Thiovulum* spp. are capable of oxidizing reduced sulfur compounds, specifically sulfide and elemental sulfur. Sulfide oxidation is evidenced by its sulfur inclusions. The amount and type of sulfur inclusions in cells is influenced by the concentrations of H_2_S and O_2_ in the environment. Typically, cells store elemental sulfur when H_2_S is abundant in the environment, and later use the intracellular reserves of sulfur when the sulfide source in the environment is depleted [10, 82]. Sulfur inclusions were also shown to form when the supply of O_2_ is limited and as a result the sulfur cannot be entirely oxidized to soluble sulfite, thiosulfate, or sulfate. Complete depletion of sulfur inclusions from cells is not likely in Movile Cave where abundant H_2_S (< 1 mM) [21]) is available continuously and where O_2_ is scarce in most habitats, and specifically in Air Bell 2 [83]. The analysis of the 6 *Thiovulum* genomes identified the SQR gene responsible for the oxidation of sulfide to elemental sulfur, which was also highly expressed in the Movile Cave transcripts. Nevertheless, the genes required for further oxidizing elemental sulfur to sulfate, via any of the known mechanisms, were not found. An exception to this is the possible oxidation of sulfite to sulfate via the intermediate adenylyl sulfate as suggested for *Thiovulum* ES, for which the gene encoding the sulfate adenylyl transferase was originally found [11] yet, according to our annotation the necessary adenylyl-sulfate reductase genes *apr* (EC1.8.4.9) or *aprA* (EC1.8.99.2) are missing from all the *Thiovulum* genomes.

Marshall et al. (2012) proposed that *Thiovulum* undergoes frequent (daily) oxic/anoxic cycles that prevent continuous accumulation of elemental sulfur in the cell. We advance three additional options by which the *Thiovulum* may avoid excessive sulfur accumulation. First, the presence of a polysulfide reductase (*nrfD*) suggests that the cells can reduce polysulfide back to sulfide (Fig. 6). In the case of the *Ca*. T. stygium strain Movile, activity of *nrfD* was confirmed by the transcriptomic data. Second, the identification of different rhodanese genes, known to be involved in thiosulfate and elemental sulfur conversion to sulfite [84], and of a sulfite exporter (*tauE*) in *Ca*. T. stygium strain Movile, all of which were found to be expressed in our samples, suggests that *Thiovulum* may oxidize elemental sulfur to sulfite and transport the latter out of the cell. The rhodanese genes found in the same predicted operon as the respiratory nitrate reductase genes (*nar*), were ca. 5-fold more expressed than a second pair of rhodanese genes. Third, we propose that cave-dwelling *Thiovulum* spp (*Ca*. T. stygium) are capable of dissimilatory nitrate reduction to ammonia (DNRA) using elemental sulfur [85] a process already shown in *Campylobacterota* (e.g., *Sulfurospirillum deleyianum*) [86]. *Ca*. T. stygium strain Movile and strain Frasassi contain not only the *nar* (*narGH*) genes for nitrate reductase, but also by the *nap* genes, coding the periplasmatic nitrate reductase known for its higher affinity and ability to function in low nitrate concentrations [87]. Additionally, they harbor the gene for the epsilonproteobacterial hydroxylamine dehydrogenase (ε*hao*). Hydroxylamine dehydrogenase is known from other *Campylobacterota* (e.g., *Campylobacter fetus* or *Nautilia profundicola*) and was shown to mediate the respiratory reduction of nitrite to ammonia [73]. The *hao* gene was not found in the genome of any of the marine *Thiovulum* spp., suggesting that the *hao* gene may not be part of the core *Thiovulum* genome. Normally, *Campylobacterota* that utilize hydroxylamine dehydrogenase do not have formate-dependent nitrite reductase, matching the annotation of *Ca*. T. stygium strain Movile and strain Frasassi. *Campylobacterota* typically use periplasmic nitrate reductase (*nap*) and do not have membrane-bound *narGHI* system [88, 89]. All marine *Thiovulum* species have only *nar* systems while the *Ca*. T. stygium strain Movile and strain Frasassi have both types, suggesting that the *nap* genes may be a later acquisition by cave-dwelling *Thiovulum*. Nevertheless, while genomic information is suggestive of the presence or absence of specific enzymes and pathways, additional experiments and gene expression data are required to determine which of the genes are utilized and under which environmental conditions. Our transcriptomic analysis points out that at the time of sampling the *nap* and ε*hao* genes were minimally expressed as compared to other sulfur and nitrogen metabolism genes (Fig. 6, Supplementary Dataset 1), suggesting that the DNRA pathway was not very active in the *Thiovulum* community at the time of sampling. In contrast, the high expression of both copies of rhodanase genes as well as the sulfite exporter (*tauE)* suggest that elemental sulfur may have been converted to sulfite and excreted.

We propose that, if O_2_ is available, sulfide is oxidized to elemental sulfur with oxygen as an electron acceptor (as may have been the case for part of the community at the time of sampling given the high expression of cytochrome *c* oxidase cbb3; Fig. 6). However, when cells are located below the O_2_ penetration depth, the Movile Cave *Thiovulum* may oxidize sulfide using NO_3_^-^ as an electron acceptor, in a process of dissimilatory nitrate reduction to ammonium, as in other *Campylobacteraceae*.

### Air Bell 2 vs. Lake Room

*Thiovulum* did not appear to be the most abundant organism in the surface water of the Lake Room either by microscopy- or DNA based observations. Yet, its rRNA made up over 94% of the transcriptomic data, similar to the RNA samples from the hypoxic Air Bell 2. Nevertheless, it is known that community profiles obtained from DNA, representing pseudo-abundance, and those from RNA, representing degree of activity, can substantially differ from each other [90, 91]. Additionally, we cannot exclude that the community composition may have shifted during the time lapse between the DNA and RNA sampling. The presence and high activity of *Thiovulum* at the surface of both the Lake Room and Air Bell 2, environments that differ significantly in the overlaying atmosphere (normoxic *vs*. hypoxic, respectively), suggest that either the *Ca*. T. stygium strain Movile is not sensitive to these differences in O_2_ concentration, or it mostly aggregates at different locations in the stagnant, oxic surface (<1 mm) of the different cave chambers.

The expression profiles differed significantly between the Lake Room and Air Bell 2, and it is evident (Fig. 6) that most genes recognizable as involved in cell metabolism had higher expression levels in Air Bell 2, though not all at statistically significant levels (Supplementary Dataset 1). More than half of the genes overexpressed by *Thiovulum* in the Lake Room could not be assigned any function, making it impossible to understand it’s specific metabolic activity in that compartment of the cave. However, the high expression of retron-type reverse transcriptase and Type II restriction enzymes in the Lake Room can be indicative of an ongoing phage infection [92, 93], which may explain the reduced metabolic activity and elevated expression of defense systems, though only one CRISPR associated gene was highly expressed. Quantification of viral transcripts showed an overall higher expression in the Lake Room (Fig. S9); however, at this stage it is not possible to directly connect these transcripts to *Thiovulum*.

### Cell motility and veil formation

*Thiovulum* sp. often forms large veils of interconnected cells. Additionally, the cells can attach to surfaces using a slime stalk secreted by the antapical organelle located at the posterior side of the cell [1, 10]. Short peritrichous filaments (Fig. 3D) observed on the surface of the cells from Movile Cave resemble those noticed earlier in *Thiovulum* species and referred to as flagella [6]. While all genes necessary for flagella assembly were found in the Movile Cave and all other *Thiovulum* sp. genomes, so were genes for type IV pili, though with closest homologs in different types of pili systems (T4Pa, T4pB, MSH). Evaluating available electron microscopy images, we suggest that future, focused studies, should evaluate whether all these filaments are indeed flagella.

Our electron microscopy images (as well as previous ones of connected *Thiovulum* cells) showed that the filamentous structures that occur all around the cell are formed in bundles and some of them are connecting cells. As these filaments are not exclusively polar, they are likely not secreted from the antapical organelle [1] the fibrillar structure of which was clearly depicted in earlier studies [10, 94]. Additionally, they are much thinner than the stalk-like structure shown by de Boer et al. [10]. These peritrichous structures around the cells are commonly regarded as flagella. The length of these structures based on de Boer et al. [10], Robertson et al. [1], and our electron microscopy data, is under 3 µm. This is much shorter than flagella that are typically >10 µm in length [95], and is closer to the 1–2 µm lengths known for pili. The diameter of the *Thiovulum* filamentous structures see in SEM images is in the range of 20–30 nm, 2–4 times larger than typical type IV pili [96] yet in the same range as reported for pili bundles [97] or seen in images of *Campylobacterota* pili [98] (which may or may not be bundled). Accordingly, bright-field scanning TEM analysis on some filaments revealed that these structures consist of multiple thinner pili with diameters <10 nm (Fig. 3). In light of these observations, we propose that at least some of these structures are pili, specifically type IV pili, for which the necessary genes were found in the genomes, and that are known, among other functions, to connect cells to surfaces or other cells [99, 100].

Petroff et al. [9] investigated the physics behind the 2-dimensional plane assembly of *Thiovulum* veils and suggested it to be a direct result from the rotational movement attracting cells to each other. Nevertheless, our SEM images showed cells that are physically attached one to the other, suggesting that several mechanisms and steps may be involved. Type IV pili retraction can generate forces up to 150 pN known to be involved in twitching motility in bacteria [99]. *Caulobacter crescentus* swims at speeds of up to 100 µm s^−1^ using a single flagellum aided by multiple pili. Thus, if coordinated, these pili may be part of the explanation of the swimming velocity of some *Thiovulum* species which is, at up to ca. 615 µm s^−1^, 5 to 10 times higher than that of other flagellated bacteria [7]. The genomic information and re-evaluation of electron microscopy data raise new questions concerning the nature of the extracellular structures on the surface of *Thiovulum* and call for new targeted investigations into this topic.

## Conclusions

Movile Cave is an ecosystem entirely depending on chemosynthesis. We showed that the planktonic microbial accumulations are dominated by *Thiovulum*, a giant bacterium typically associated with photosynthetic microbial mats. We further showed that *Thiovulum* dominates the active fraction in surface waters of hypoxic and normoxic compartments of the cave, suggesting an ability to adapt to different O_2_ concentration, by means that remain to be studied.

Our results highlight the existence of a clade of cave and subsurface *Thiovulaceae* that based on genomic information differs significantly from marine *Thiovulum*. The genomes of two conspecific cave strains from Movile and Frasassi caves, included here in the new species *Candidatus* Thiovulum stygium, suggest that these bacteria can perform dissimilatory reduction of nitrate to ammonium, when O_2_ is unavailable. Thus, *Thiovulum* may play a role in the nitrogen cycle of sulfidic caves, providing readily available ammonia to the surrounding microorganisms. The coupling of DNRA to sulfide oxidation provides a direct and more productive source of ammonium.

This investigation of six *Thiovulum* genomes, coupled with observations of current and previous microscopy images, questions the number of flagella *Thiovulum* cells have, suggesting that many of the peritrichous filamentous structures around the cells are in fact type IV pili that may be involved in rapid movement and cell-to-cell interactions.

The collective behavior of *Thiovulum* is still a puzzle and there may be more than one mechanism keeping the cells connected in clusters or in veils. Our SEM images suggest the cells are connected by thread-like structures. Petroff et al. [9] showed, on another strain, that there is no physical connection between the cells, suggesting that the swimming behavior of individual cells is what keeps them together. More research is therefore needed to understand if these different mechanisms are driven by strain variability or by different environmental conditions.

## Supplementary information

Supplementary Material

Figure S3 High Resolution

Figure S4 High Resolution

Figure S5 High Resolution

Figure S6 High Resolution

Figure S7 High Resolution

Figure S8 High Resolution

Supplementary dataset 1

Supplementary dataset 2

Supplementary dataset 3

Supplementary dataset 4

Supplementary dataset 5

Supplementary dataset 6

Supplementary dataset 7

Supplementary video

## Data Availability

All sequenced data from Movile Cave is available under bioproject accession number PRJNA673084. The genome of *Thiovulum* sp. from Frasassi is available under bioproject accession number PRJNA846597. The genomes of *Ca*. T. imperiosus, and *Ca*. T. karukerense are available under bioproject accession number PRJNA830902. All other data are provided as part of this manuscript and its supplementary material, including annotation results of all genomes that are provided as supplementary datasets.
