# Supplementary material for: Cave Thiovulum (Candidatus Thiovulum stygium) differs metabolically and genomically from marine species
Source: ISME J. 2022 Dec 17;17(3):340–53. doi: 10.1038/s41396-022-01350-4 (PMC9938260; doi:10.1038/s41396-022-01350-4)
Supplement: Supplementary file 1 — Supplementary Material [file 41396_2022_1350_MOESM1_ESM.pdf]

Additional methods used for water chemistry

Measurements of Mn were obtained as part of the cave monitoring conducted by some of the co-authors and were analyzed using ion chromatography as previously described [71] Dissolved inorganic carbon (DIC) was measured as alkalinity on site from an unfiltered sampled using the methyl-orange titrimetric method using a micro-dosimeter. For dissolved organic carbon (DOC) measurements 50 mL samples (triplicates) water samples were filtered on site, using a 0.45 µm pore size filter and single use syringe into acid-washed opaque HDPE bottles. In the laboratory, the samples were transferred to opaque glass bottles and upon adjusting their pH to 2 using 2M HCl, the samples were stored at 4 °C till further analysis on a total organic carbon analyzer (Multi N/C2100S Analyzer, Analytik Jena, Jena, Germany). For the analysis the sample was further acidified with 0.1 mL 2M HCl and flushed with oxygen. H_2S was measured with an Unisense H_2S microsensor, connected to a Unisense field microsensor meter (Unisense A/S, Aarhus, Denmark).
Methane (CH_4) was determined from triplicate samples collected in July 2022 using the head-space method using a Shimadzu 14A gas chromatography equipped with a flame ionization detector as described in [72]. Prior to analysis, water samples collected from the cave were fixed with a pellet of NaOH and kept inverted in the dark till they were analyzed two weeks later.

Table S1. Additional chemical parameters of the water in the Lake Room of Movile Cave

	**1 m (~inflow)**	**0.1 m**
**DOC**	11.1 mg/L	9.3 mg/L
**DIC**	87.6 mg/L	87.2 mg/L
**PO_4^{3-}**	0.73 µM	0.65 µM
***Fe_{tot}**	NA	2.1 µM
**Mn**	294 µM	286 µM
**CH_4**	205 µM	175 µM

* Fe data ware not determined in 2021 and are reported from previous unpublished measurements by Sarbu, S. and Popa, R.

Table S2. Single copy marker genes missing from the genomes of the 6 compared *Thiovulum* spp

Protein_ Family	T._Frasassi	T._Movile	T._ES	T._Tui_Malila	T._imperiosus	T._karukerense		Protein_ Family	T._Frasassi	T._Movile	T._ES	T._Tui_Malila	T._imperiosus	T._karukerense
PF02464.12	0	0	0	0	0	0		PF03947.13	1	1	1	0	1	1
PF12344.3	0	0	0	0	0	0		PF00238.14	1	1	1	0	1	2
PF08459.6	0	0	0	0	0	0		PF00252.13	1	1	1	0	1	1
TIGR03423	0	0	0	0	0	0		PF00189.15	1	1	1	0	1	1
PF00308.13	0	0	0	0	0	0		PF00237.14	1	1	1	0	1	1
PF04085.9	0	0	0	0	0	0		PF00673.16	1	1	1	0	1	1
TIGR00615	0	0	0	0	0	0		TIGR01574	1	1	1	0	1	1
TIGR02124	0	0	0	0	0	0		PF00831.18	1	1	1	0	1	1
PF08299.6	0	0	0	0	0	0		PF01687.12	1	1	1	0	1	1
TIGR00196	0	0	1	1	1	1		PF00281.14	1	1	1	0	1	1
TIGR00539	0	0	1	1	1	1		TIGR01087	1	1	1	0	1	1
TIGR00121	0	0	1	1	1	1		TIGR00478	1	1	1	0	1	1
PF03853.10	0	0	1	1	1	1		TIGR00233	1	1	1	0	1	1
PF04560.15	0	1	1	0	1	1		PF03631.10	1	1	1	0	1	1
PF00298.14	0	1	1	0	1	1		PF00203.16	1	1	1	0	1	1
PF03946.9	0	1	1	0	1	1		PF01197.13	1	1	1	0	1	1
TIGR00922	0	1	1	0	1	1		TIGR00019	1	1	1	0	1	1
TIGR00962	0	1	1	1	1	2		TIGR01499	1	1	1	0	1	1
PF04563.10	0	1	1	1	1	1		TIGR00096	1	1	1	0	1	1
PF00542.14	0	1	1	1	1	1		PF00410.14	1	1	1	0	1	1
PF00623.15	0	1	1	1	1	1		PF01176.14	1	1	1	0	1	1
PF04983.13	0	1	1	1	1	1		TIGR01079	1	1	1	0	1	1
PF10385.4	0	1	1	1	1	1		PF00625.16	1	1	1	0	1	1
PF04998.12	0	1	1	1	1	1		PF00347.18	1	1	1	0	1	1
PF04565.11	0	1	1	1	1	1		PF02882.14	1	1	1	0	1	1
PF00562.23	0	1	1	1	1	1		PF00763.18	1	1	1	0	1	1
PF05000.12	0	1	1	1	1	1		PF00366.15	1	1	1	0	1	1
PF00466.15	0	1	1	1	1	1		PF06574.7	1	1	1	0	1	1
PF00231.14	0	1	1	1	1	2		PF13603.1	1	1	1	1	0	1
PF00213.13	0	1	1	1	1	2		PF02843.11	1	1	1	1	0	1
PF04997.7	0	1	1	1	1	1		PF02844.10	1	1	1	1	0	1
PF00687.16	0	1	1	1	1	1		PF01071.14	1	1	1	1	0	1
PF00861.17	1	0	1	0	1	1		TIGR01670	1	1	1	1	1	0
PF02768.10	1	0	1	0	1	1		TIGR00392	1	1	1	1	1	0
TIGR03002	1	0	1	0	1	1		PF02686.10	1	1	1	1	1	0
TIGR00499	1	0	1	1	1	1		PF01043.15	1	1	1	1	1	0
PF02021.12	1	0	1	1	1	1		PF00707.17	1	1	1	1	1	0
PF02606.9	1	0	1	1	2	1		PF00120.19	1	1	1	1	1	0
TIGR01082	1	0	1	1	1	1		TIGR00418	1	1	1	1	1	0
PF00712.14	2	0	1	1	1	1		PF03951.14	1	1	1	1	1	0
TIGR02350	1	1	0	1	1	0		PF03840.9	1	1	1	1	1	0
TIGR03723	1	1	0	1	1	1		PF05198.11	1	1	1	1	1	0

Supplementary Figures

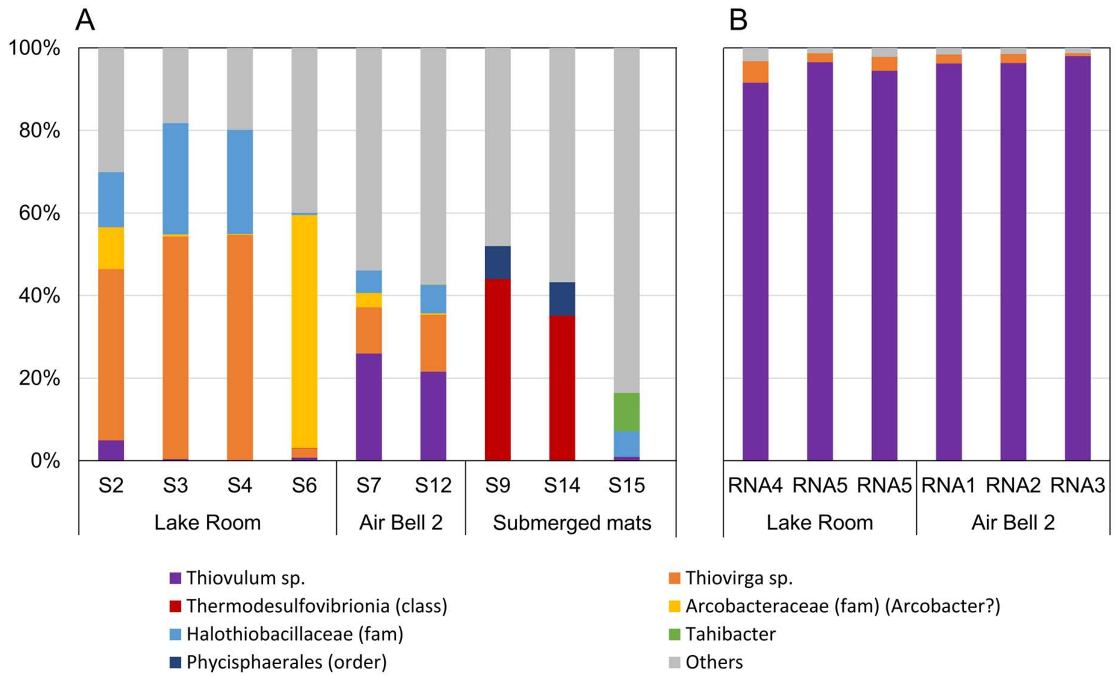

Figure S1. Community composition of water surface from the Lake Room, submerged microbial mats in Air Bell 1 and water surface in Air Bell 2 (A). The community was derived by extracting and annotating 16S rRNA genes from metagenomic data from the various samples. A similar analysis conducted on RNA samples obtained from surface water collected in the Lake Room and Air Bell 2 is showed in panel B.

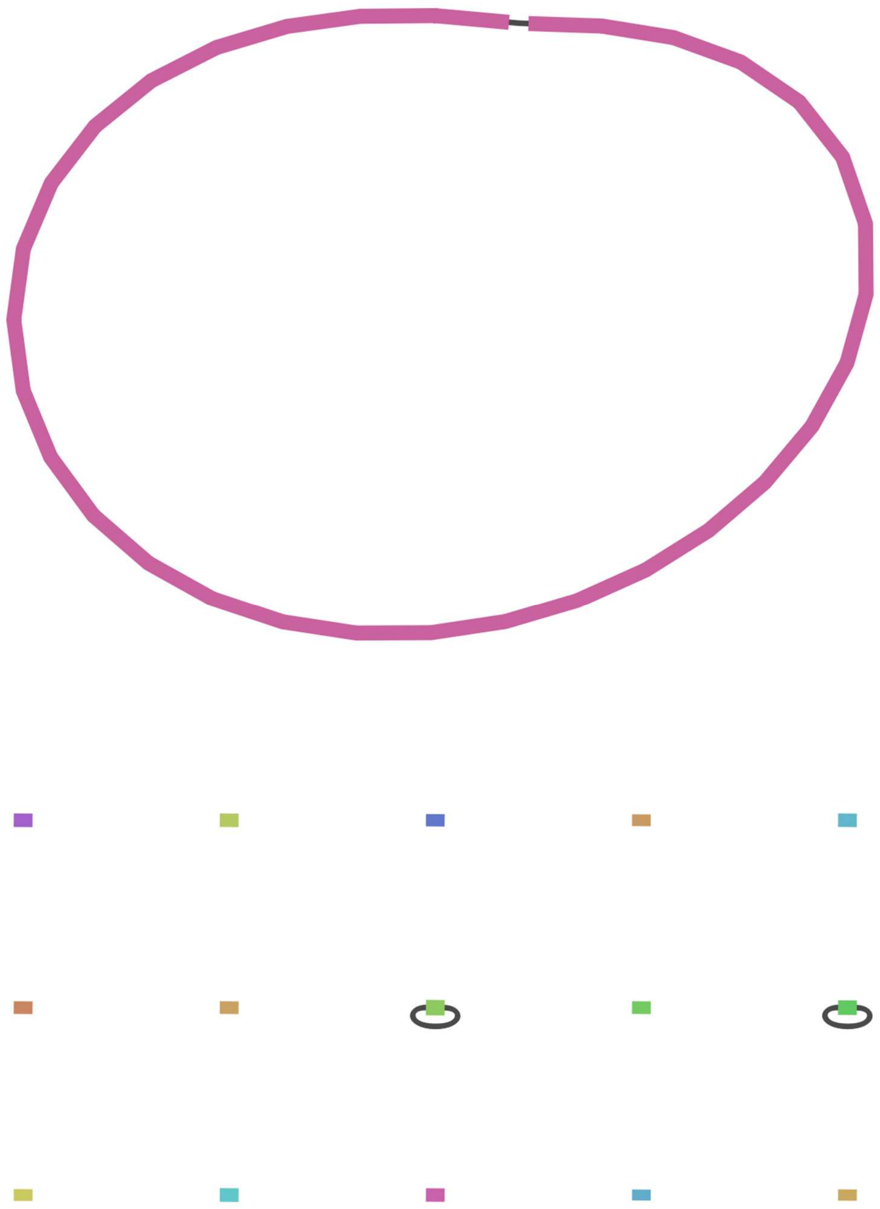

Figure S2. Assembly graph analysis using the Bandage tool, showing that the final genome of *Ca*. Thiovulum stygium genome consisted of one circular DNA sequence.

- Figures S3-S8 are available also separately at higher resolution.

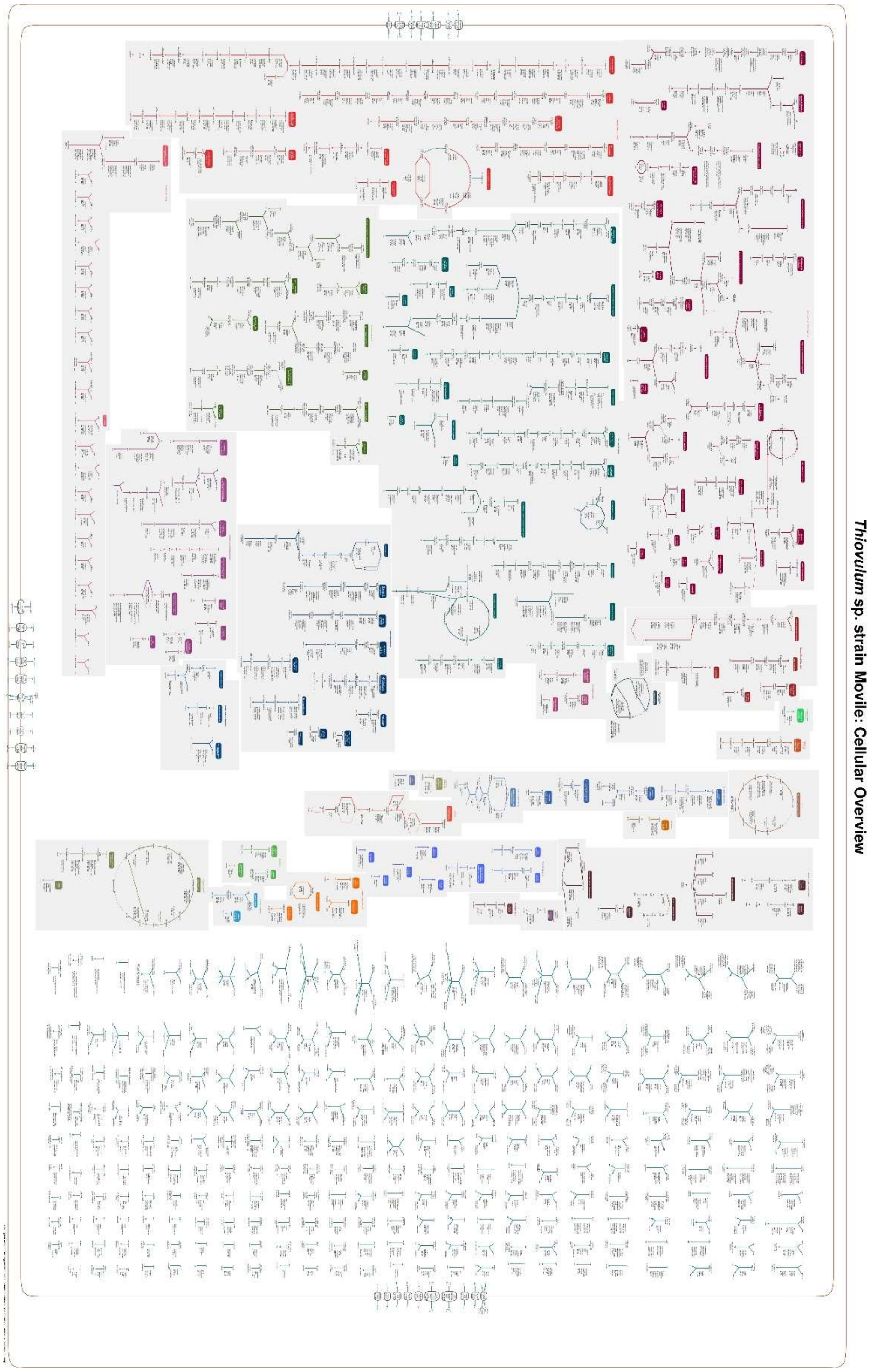

Figure S3. Overview of *Ca.* Thiovulum stygium strain Movile metabolism as generated by the Pathway Tools cell overview.

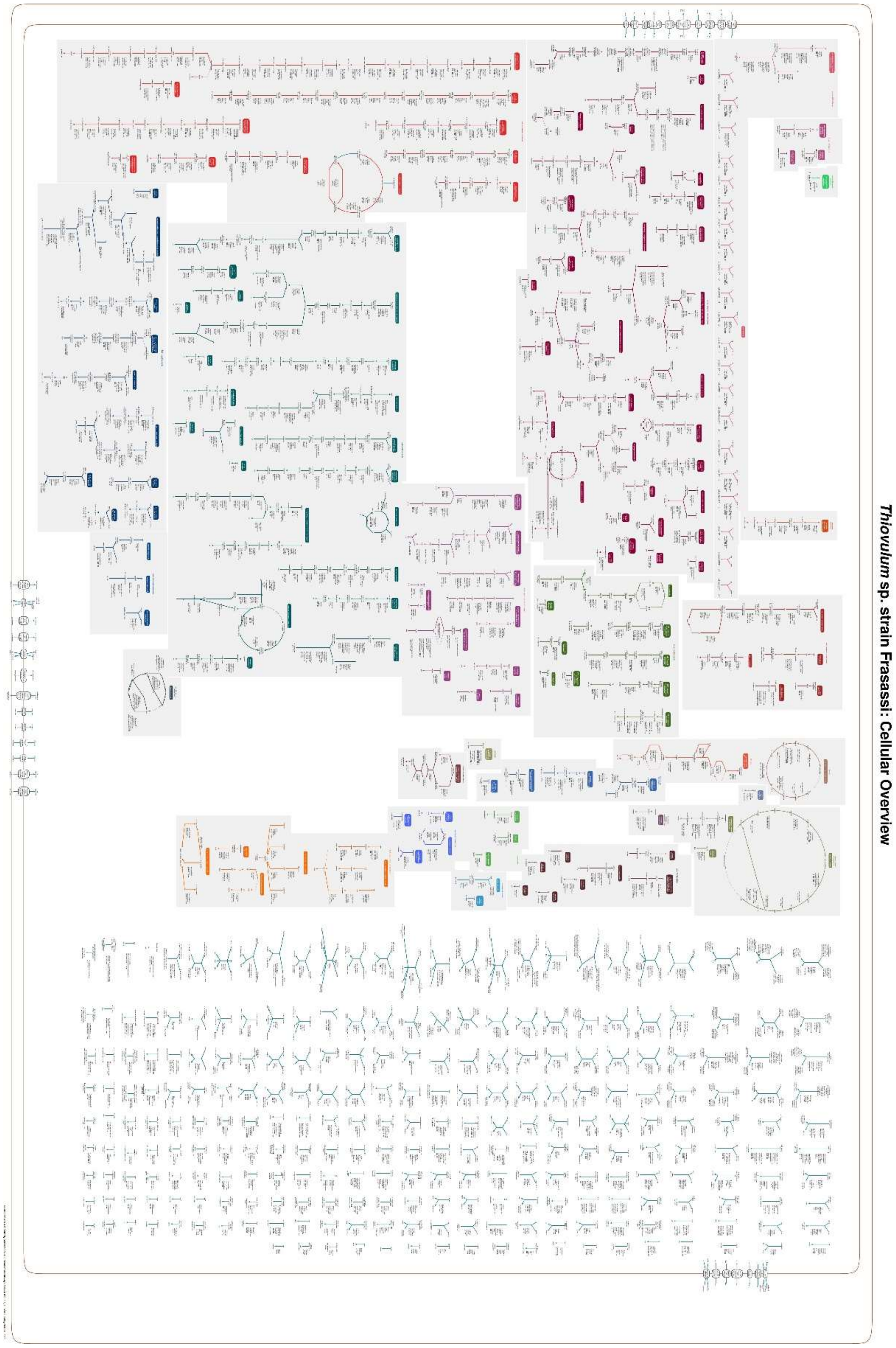

Figure S4. Overview of *Ca.* Thiovulum stygium strain Frasasssi metabolism as generated by the Pathway Tools cell overview.

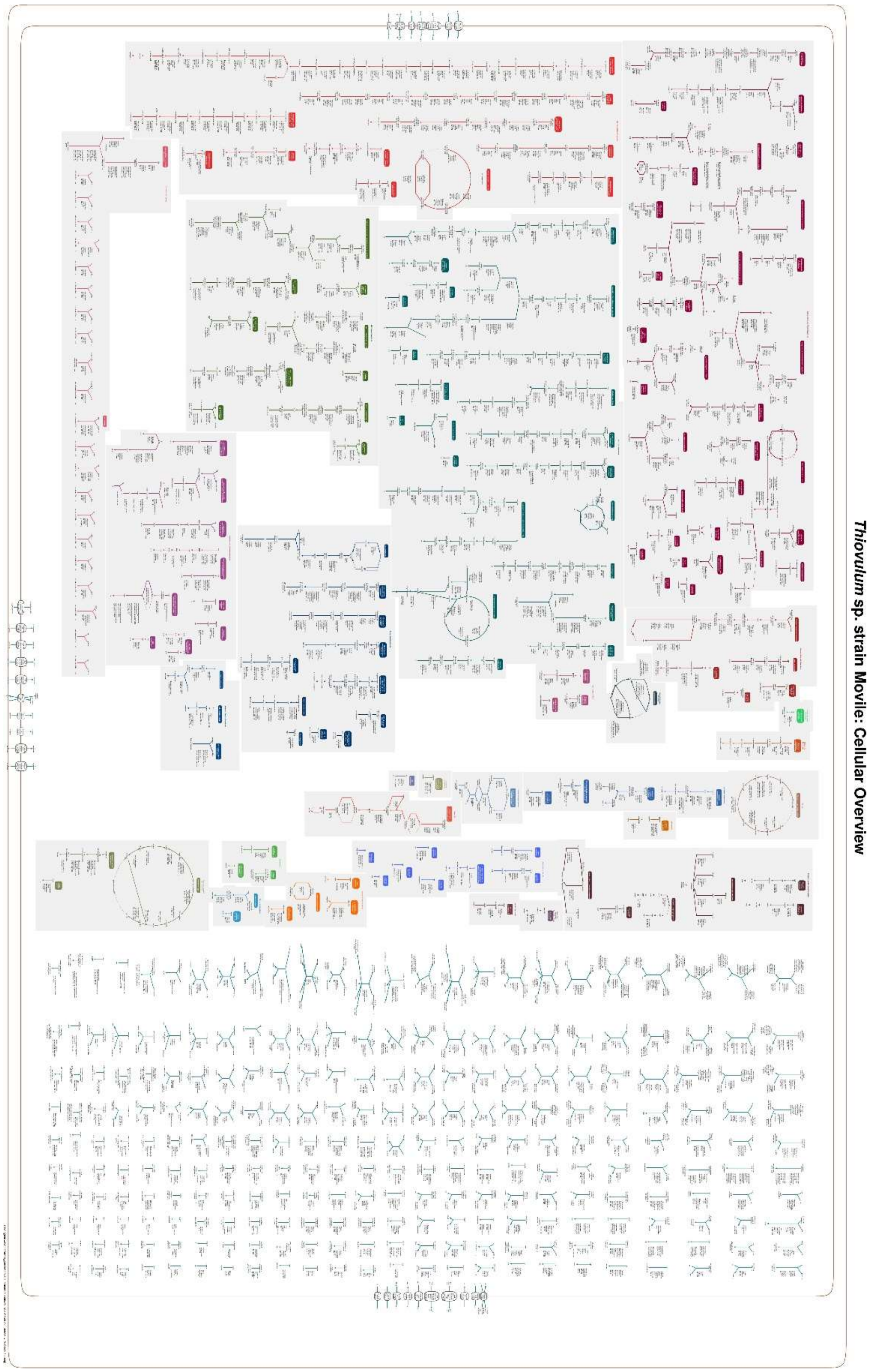

Figure S5. Overview of *Thiovulum* sp. ES metabolism as generated by the Pathway Tools cell overview.

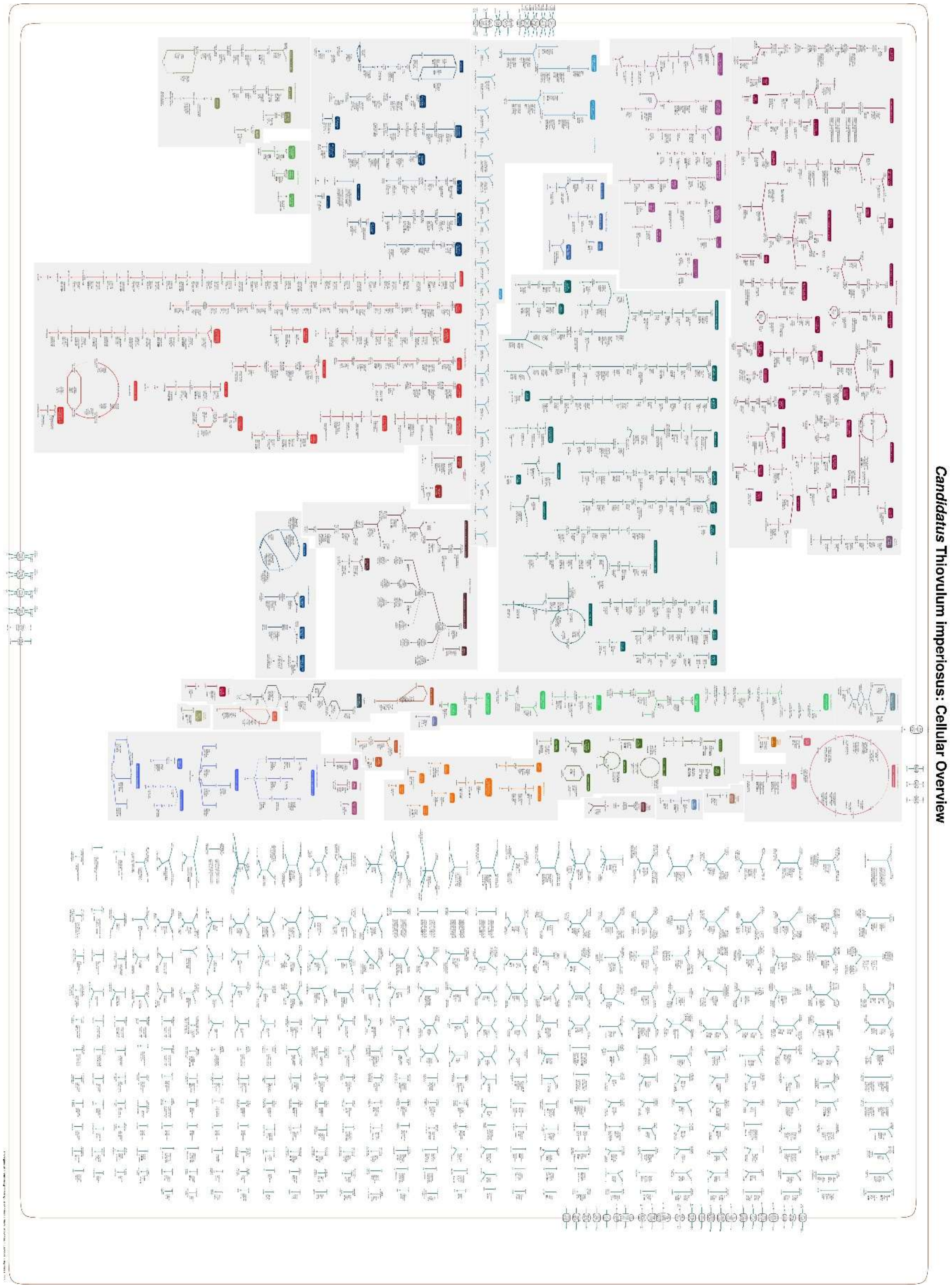

Figure S6. Overview of *Ca.* Thiovulum imperiosus metabolism as generated by the Pathway Tools cell overview.

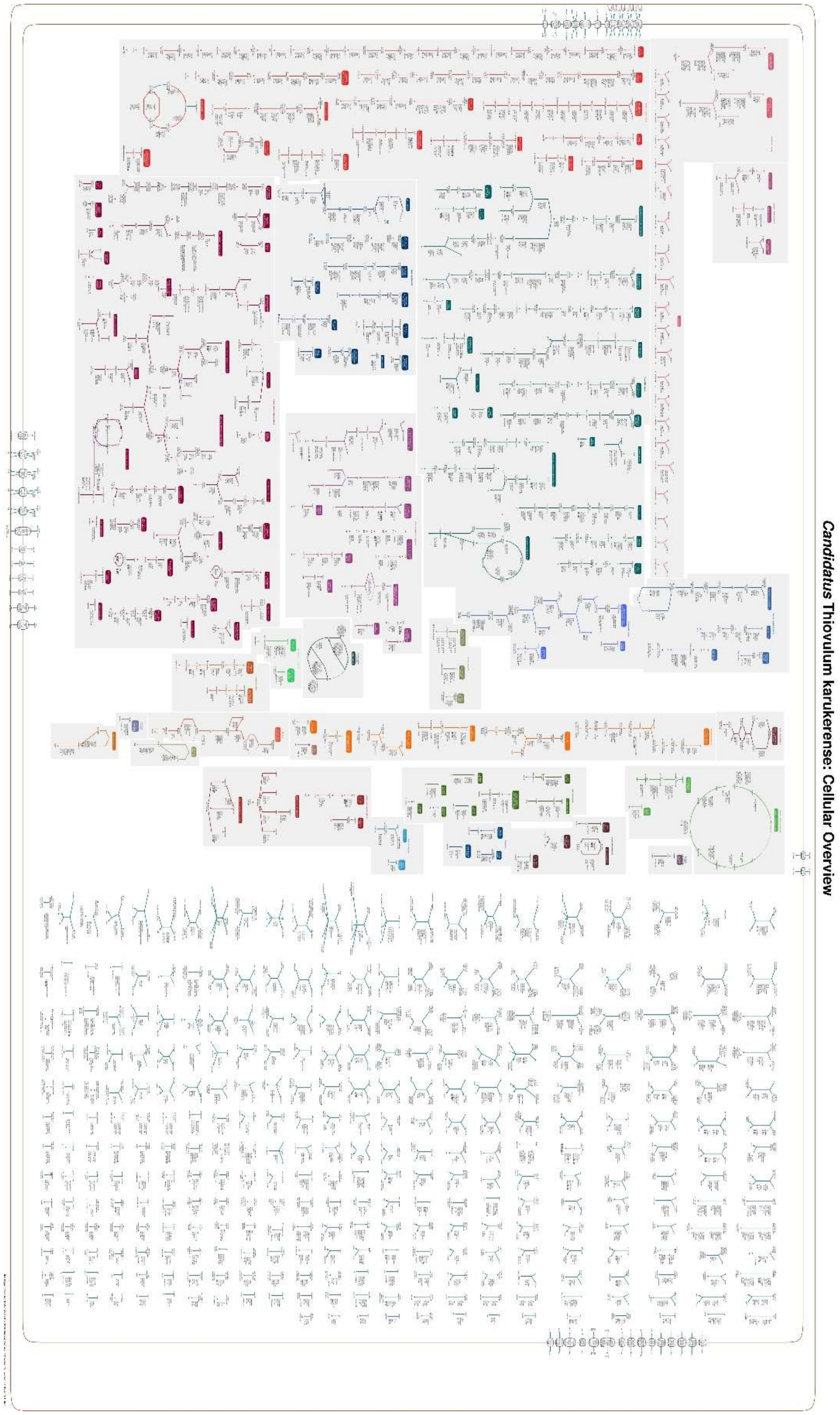

Figure S7. Overview of *Ca.* Thiovulum karukerense metabolism as generated by the Pathway Tools cell overview

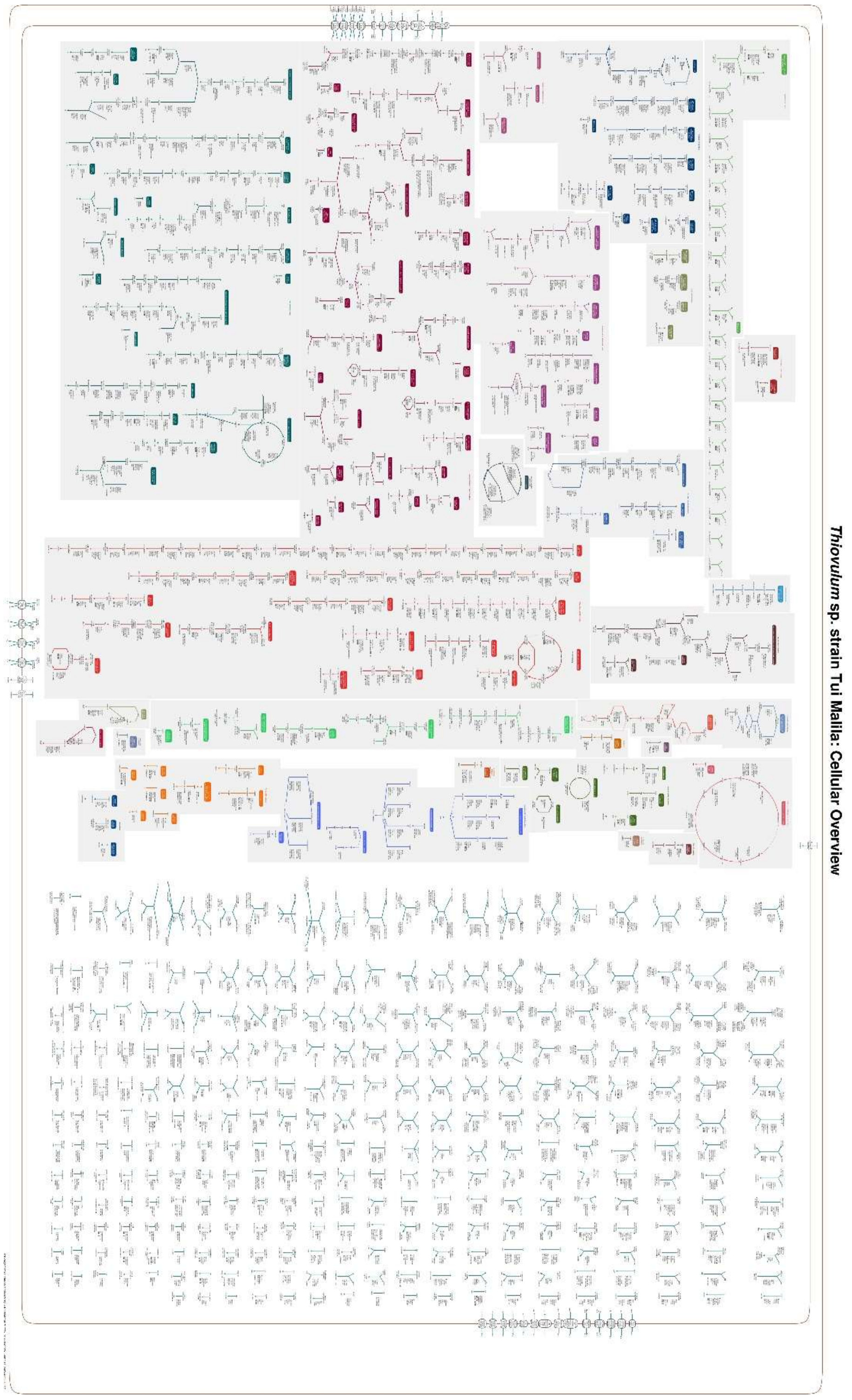

Figure S8. Overview of *Thiovulum* sp. Tui Malila metabolism as generated by the Pathway Tools cell overview

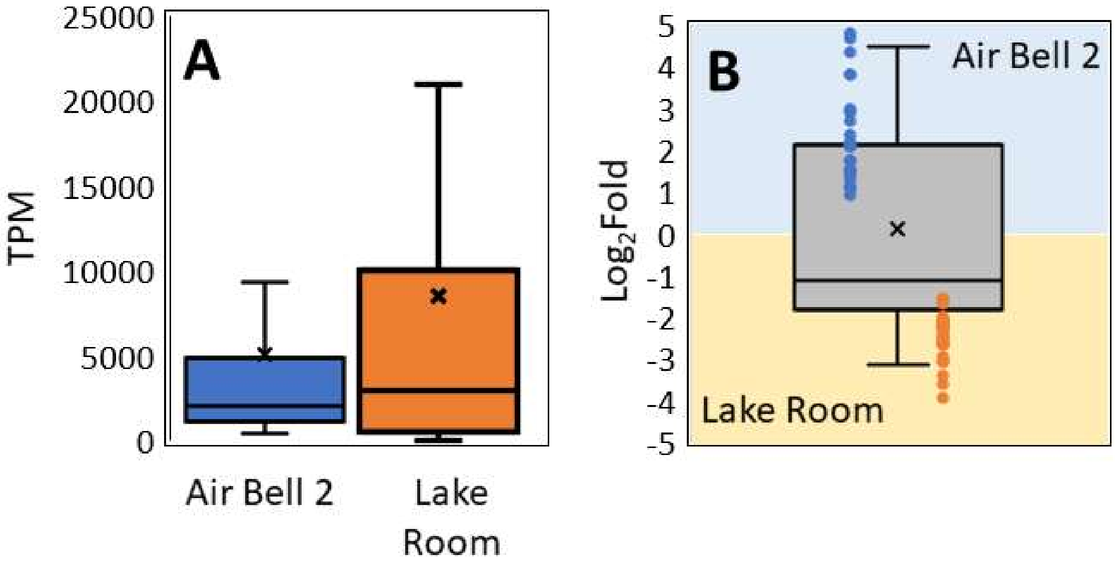

Figure S9. Quantification of reads from Air Bell 2 and the Lake Room mapping to viral transcripts expressed as transcripts per million (TPM) (A). Log 2-fold expression of viral transcripts in Air Bell 2 vs. the Lake Room. Values above 1 and below -1 show a 2-fold change in expression in favor of Air Bell 2 and the Lake Room, respectively (B). The average line in panel B, close to a value of -1 highlights the higher expression of viral transcripts in the Lake Room.

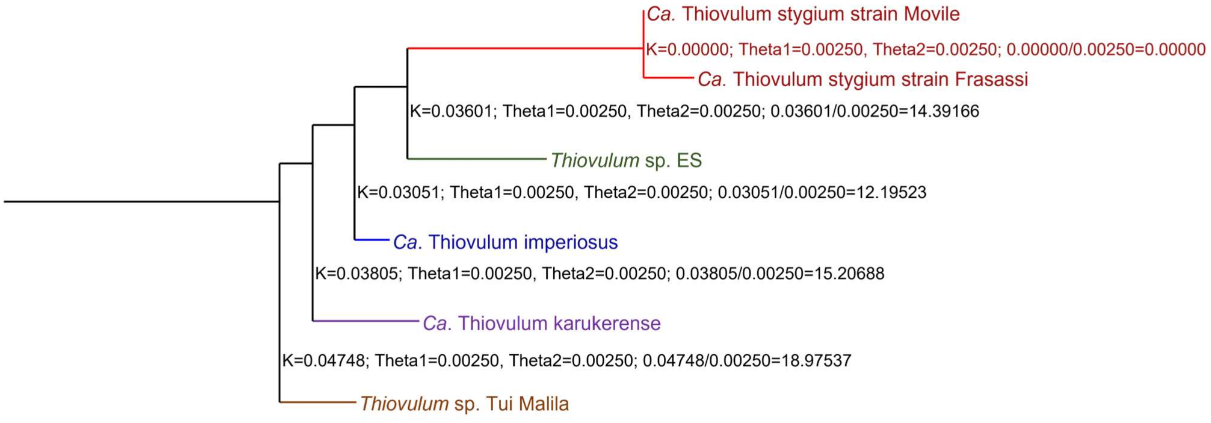

Figure S10. An excerpt of a Neighbor Joining tree constructed from the same multilocus alignment used for Fig. 3B and used for K θ / with the KoT online server (Spöri *et al.*, 2022; Birky *et al.*, 2010; Birky and Maughan, 2021). A K/θ>6 coinciding with a p=0.01 was used to delineate species, colored here with different colors.

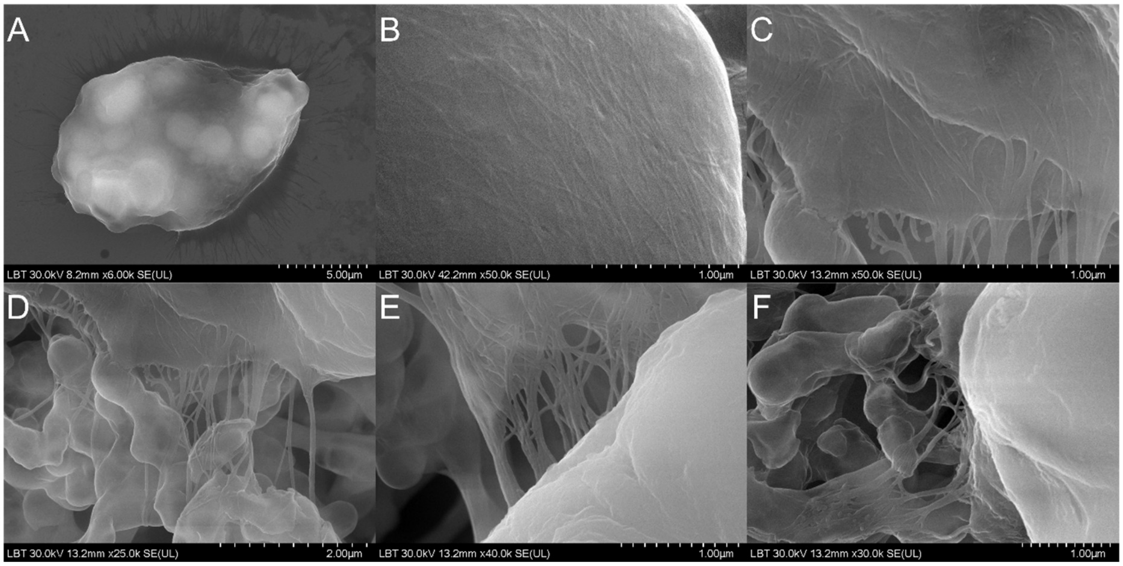

Figure S11. Electron microscopy images showing the large number and overall structure of the filaments all around cells of the Movile cave *Thiovulum* sp. strain. A) An entire cell with visible sulfur globules as well as the surrounding filaments. B) Filaments settled on the cells in absence of aqueous medium or filter to attach to. C-F) Close-up images on filaments and filament bundles attached to the surface of a polycarbonate filter. As shown in Fig. 3, the individual filaments seen here are bundles themselves, consisting of several thinner filaments of ca. 10 nm in diameter.

References

Birky CW, Adams J, Gemmel M, Perry J. (2010). Using population genetic theory and DNA sequences for species detection and identification in asexual organisms. *PLoS One* **5**: e10609.

Birky CW, Maughan H. (2021). Evolutionary genetic species detected in prokaryotes by applying the K/θ ratio to DNA sequences. *bioRxiv* 2020.04.27.062828.

Spöri Y, Stoch F, Dellicour S, Birky CW, Flot J-F. (2022). KoT: an automatic implementation of the K/θ method for species delimitation. *bioRxiv* 2021.08.17.454531.
