# Supplementary figures and images for: Cave Thiovulum (Candidatus Thiovulum stygium) differs metabolically and genomically from marine species
Source: ISME J. 2022 Dec 17;17(3):340–53. doi: 10.1038/s41396-022-01350-4 (PMC9938260; doi:10.1038/s41396-022-01350-4)

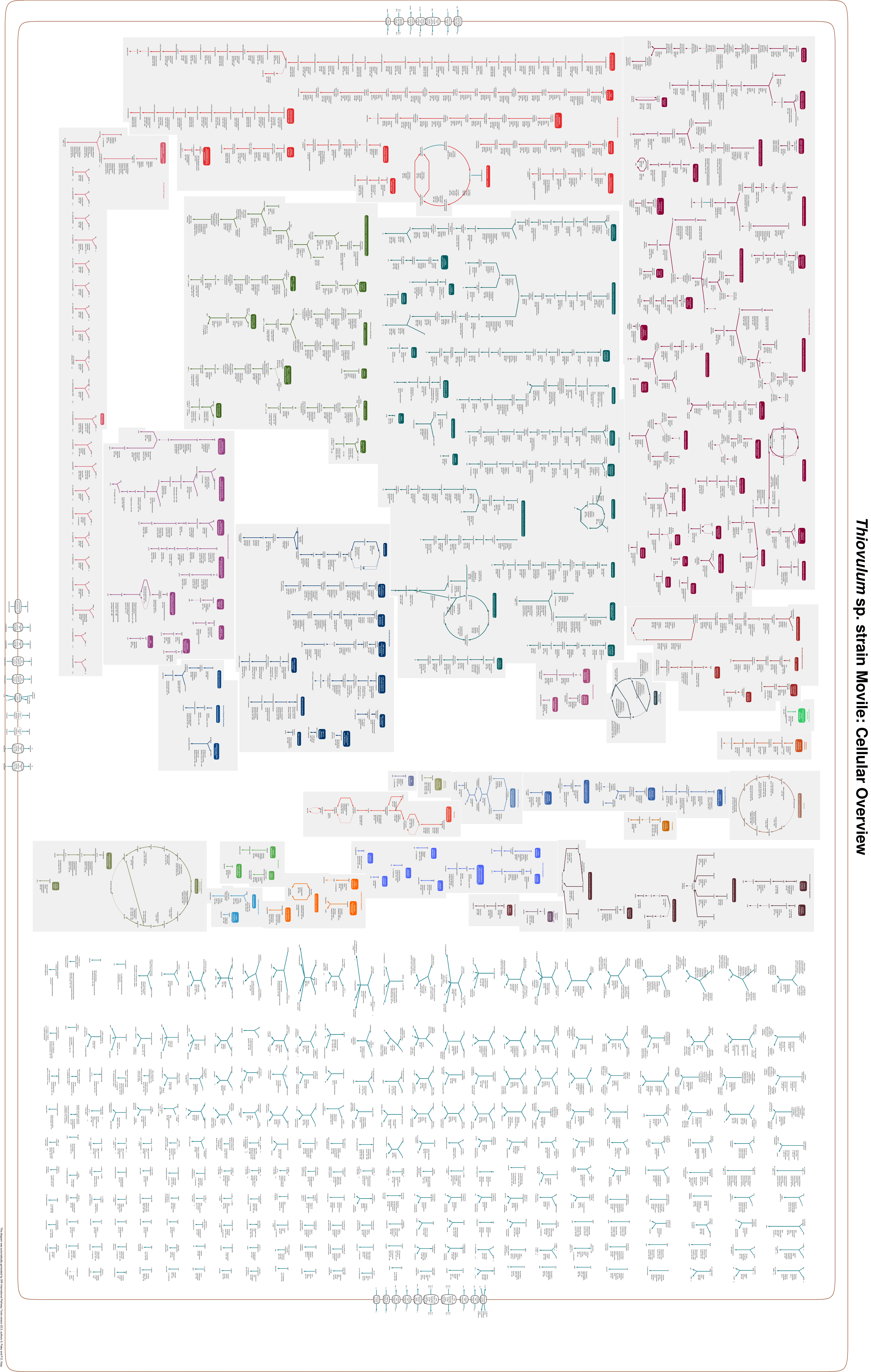

Supplement: Supplementary file 2 — Figure S3 High Resolution [file 41396_2022_1350_MOESM2_ESM.jpg]

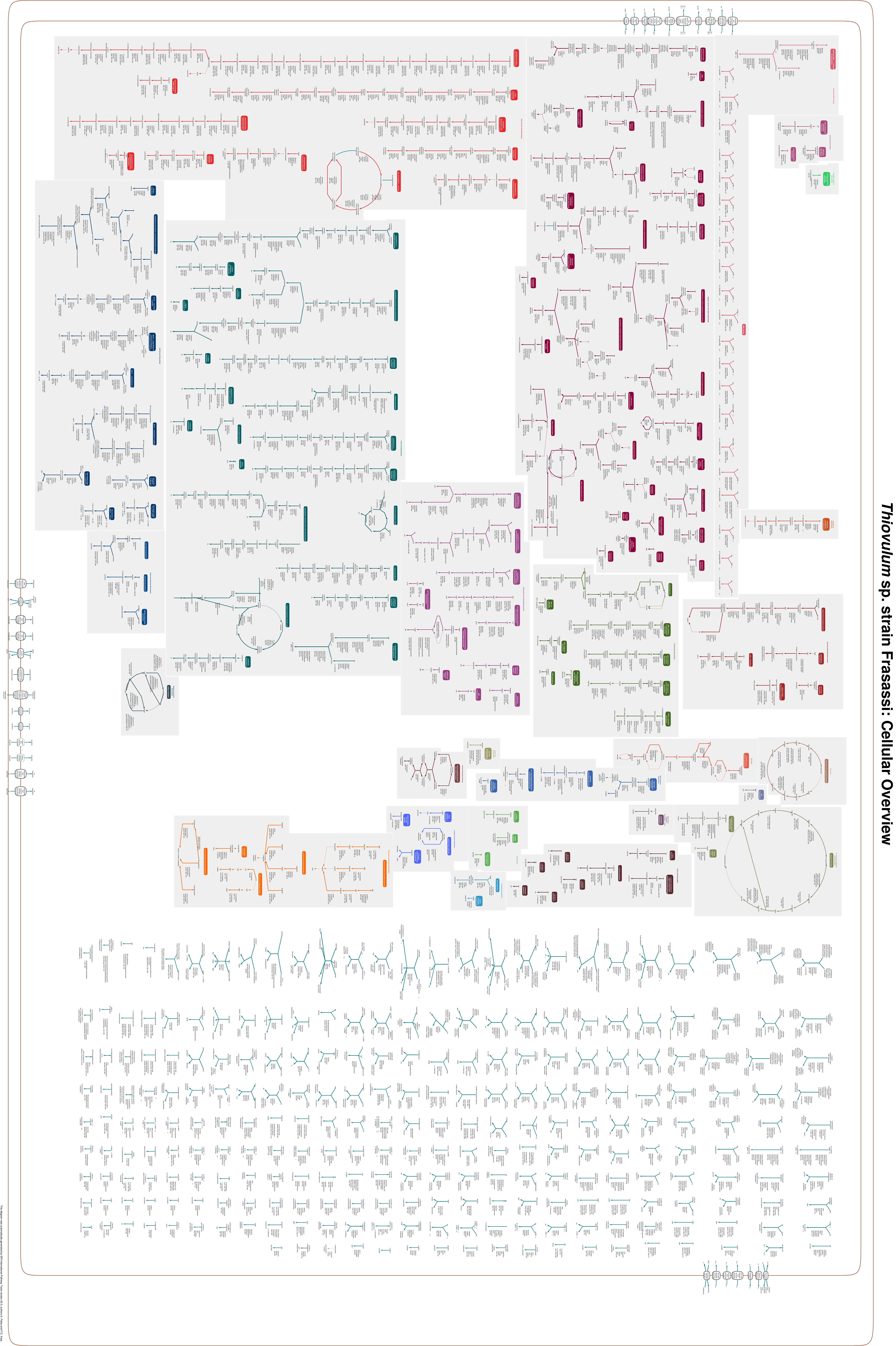

Supplement: Supplementary file 3 — Figure S4 High Resolution [file 41396_2022_1350_MOESM3_ESM.jpg]

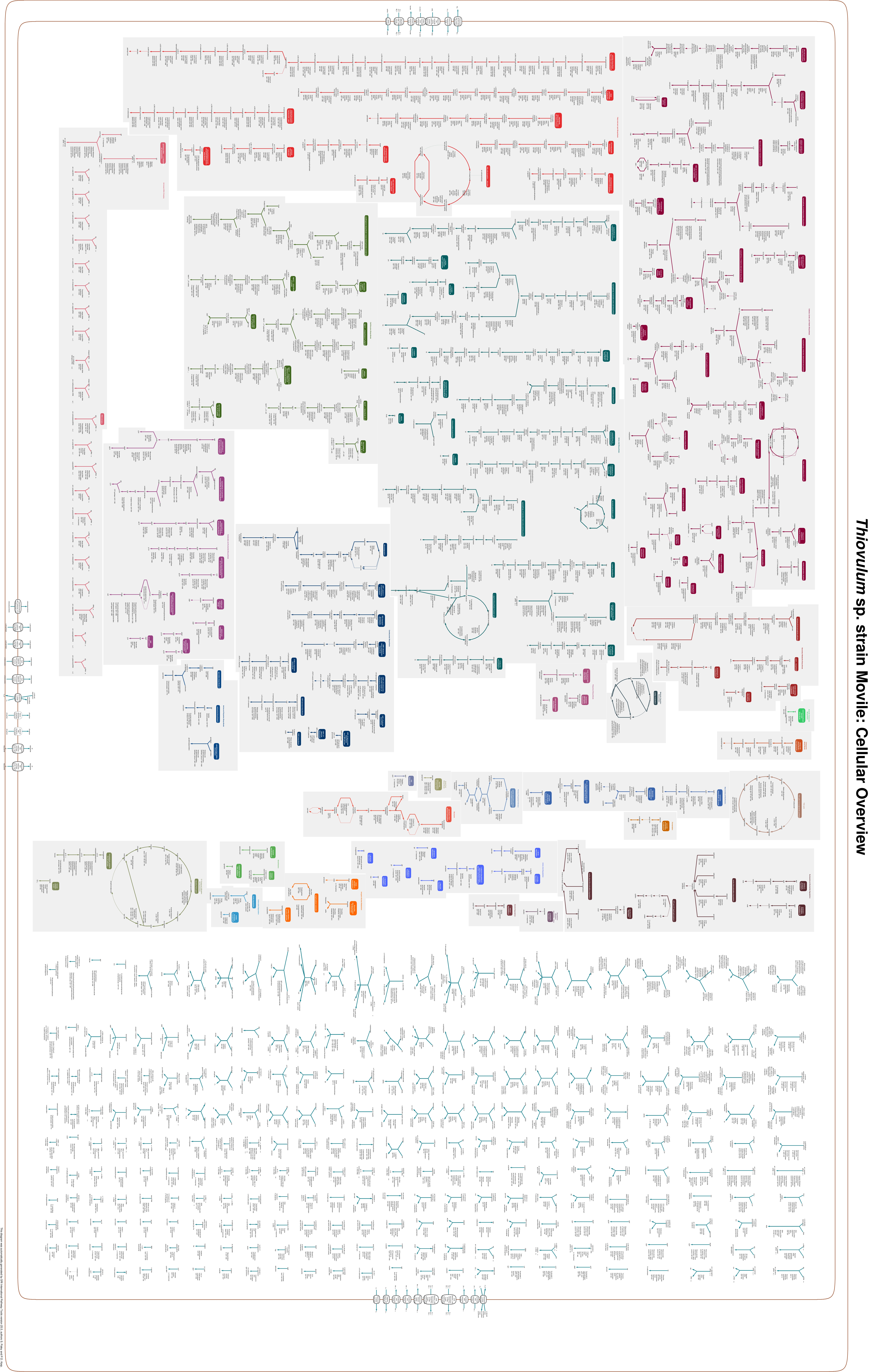

Supplement: Supplementary file 4 — Figure S5 High Resolution [file 41396_2022_1350_MOESM4_ESM.jpg]

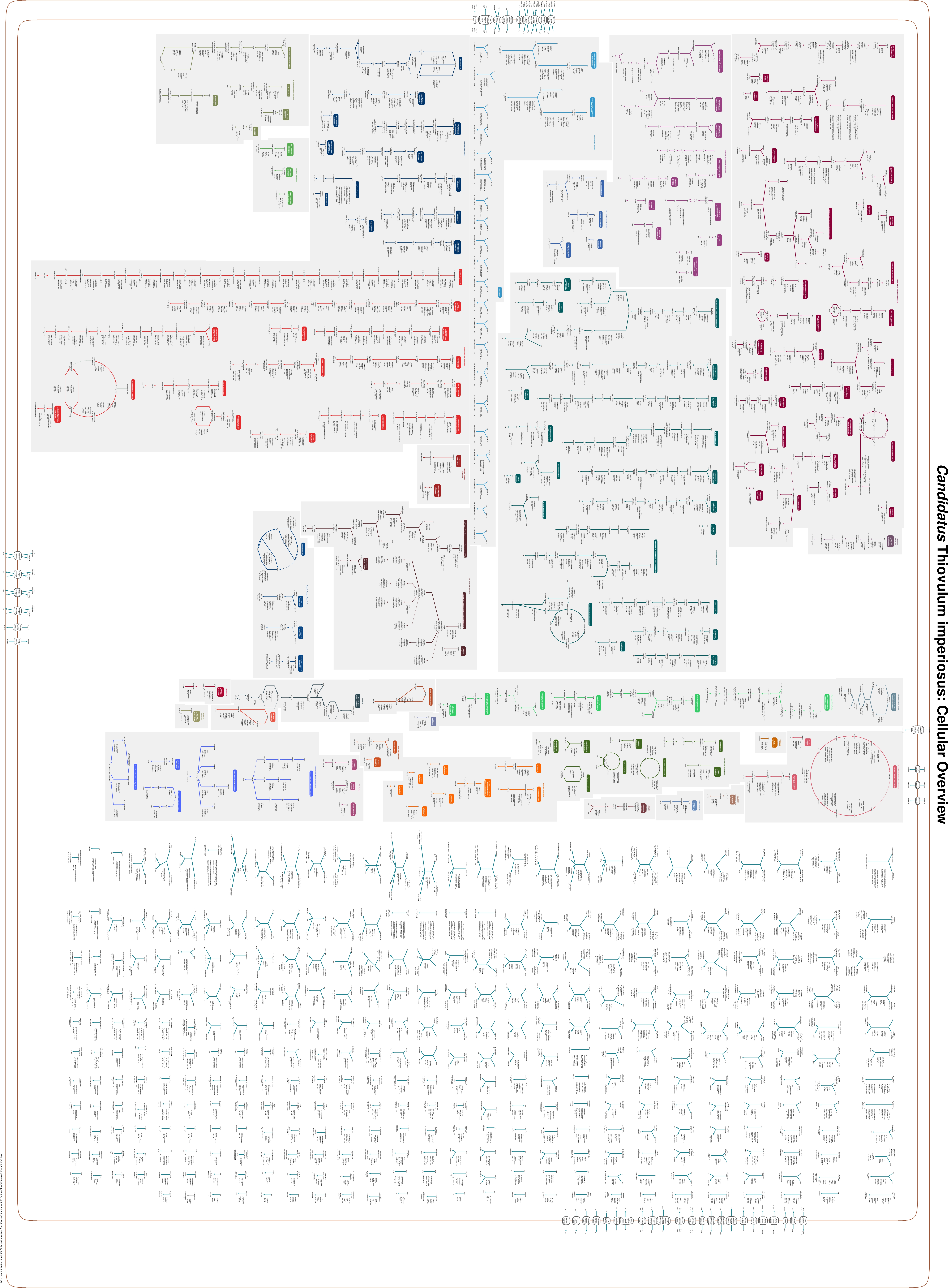

Supplement: Supplementary file 5 — Figure S6 High Resolution [file 41396_2022_1350_MOESM5_ESM.jpg]

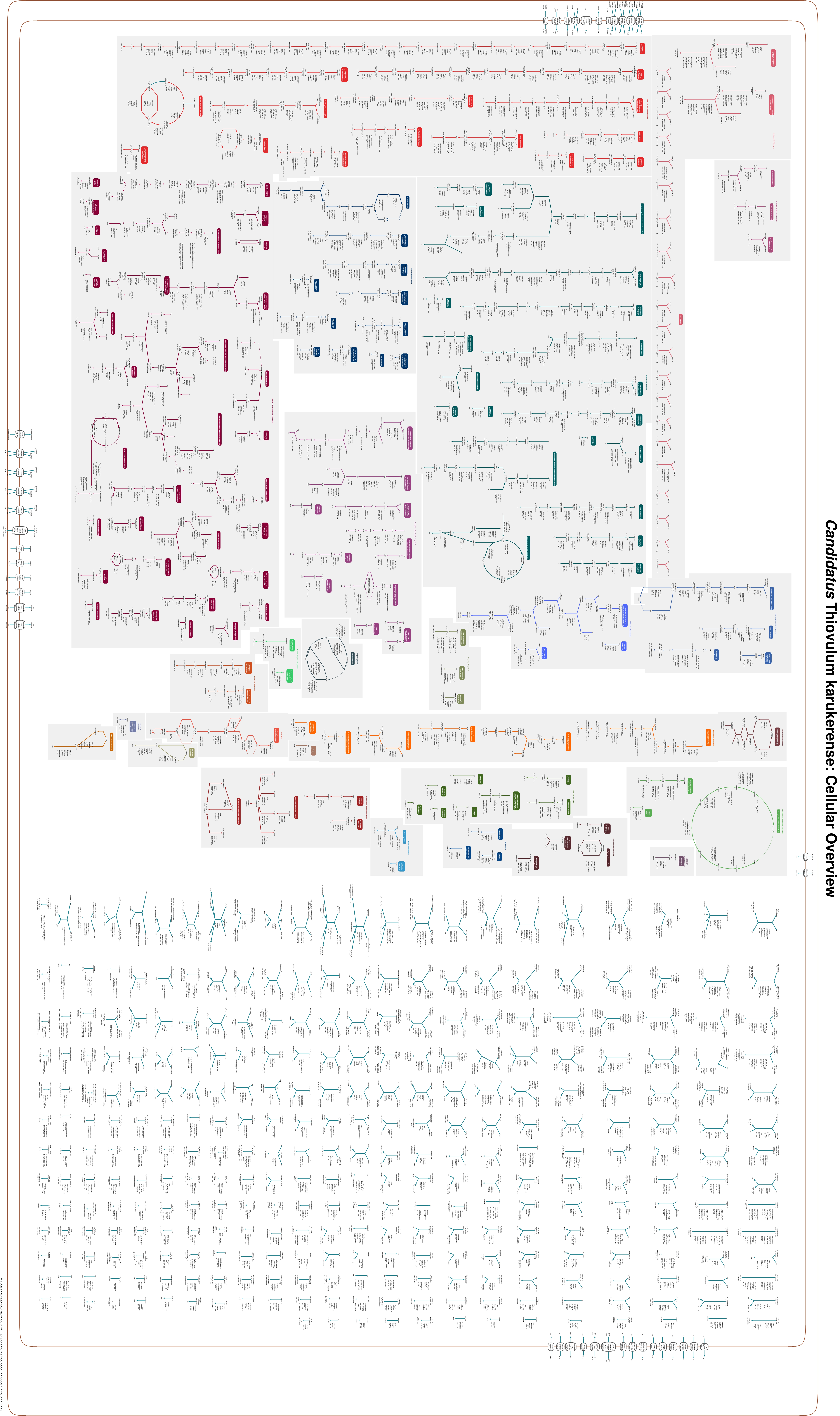

Supplement: Supplementary file 6 — Figure S7 High Resolution [file 41396_2022_1350_MOESM6_ESM.jpg]

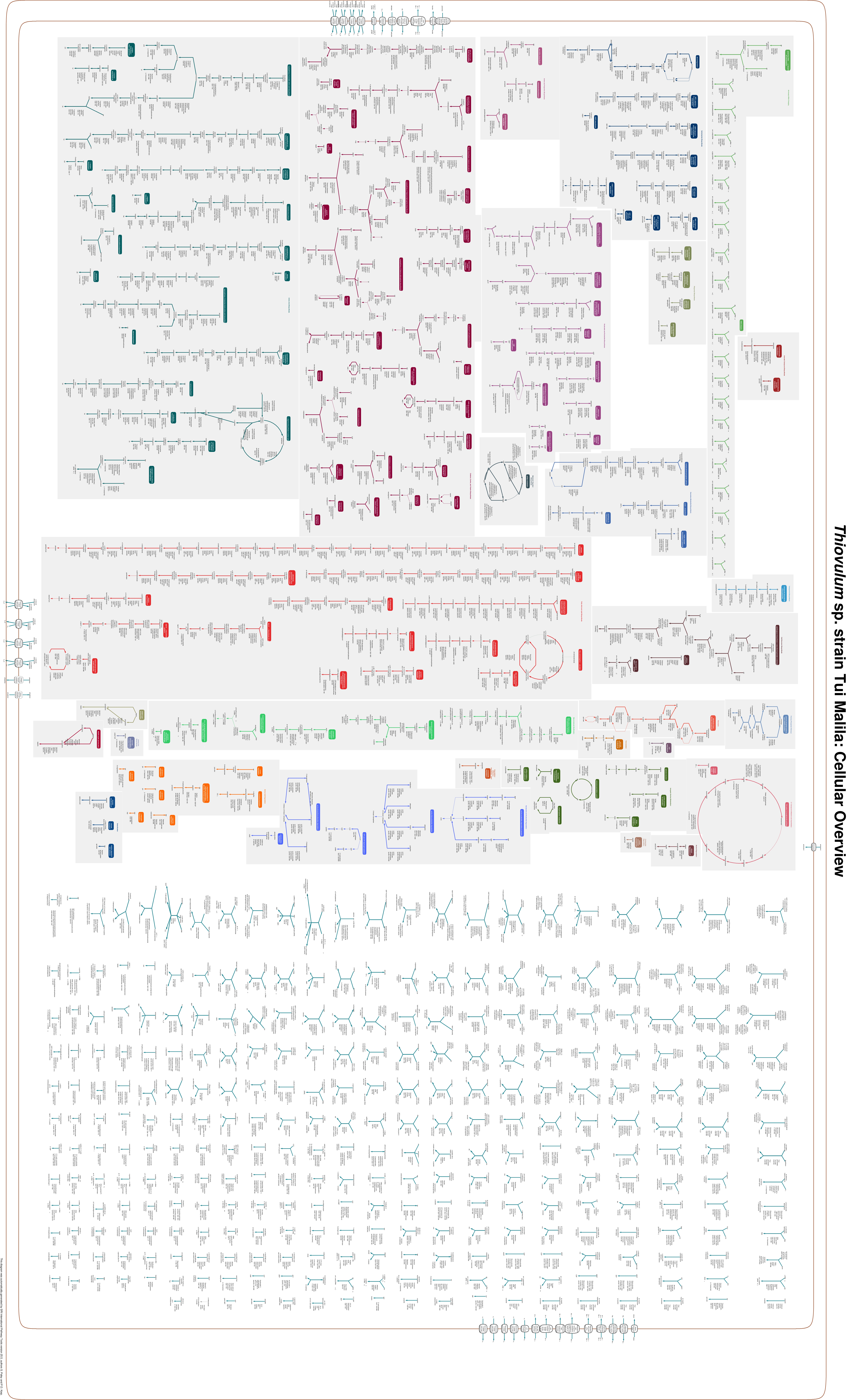

Supplement: Supplementary file 7 — Figure S8 High Resolution [file 41396_2022_1350_MOESM7_ESM.jpg]
